# An expert patient program for people living with multiple sclerosis improves knowledge and empowerment: a pre-test, post-test multicenter implementation study

**DOI:** 10.3389/fneur.2025.1581038

**Published:** 2025-04-29

**Authors:** Miguel Ángel Robles-Sánchez, Paloma Amil-Bujan, Cristina Bosch-Farré, Clàudia Coll-Martínez, María Jesús Arévalo, Elisenda Anglada-Clofent, Rebeca Menéndez-Díaz, Montserrat Aroca-Alsina, Miguel Merchan-Ruiz, Santiago Pérez-Hoyos, Ángel Guillermo Arévalo-Bernabé, Xavier Montalban, Ana Hernando, Sara LLufriú, Silvia Peralta-Moncusí, Lluís Brieva, Cristina Ramo-Tello, Mariona Carabí, Nuria Solà-Valls, Maria Cinta Zabay, Maria Alba Mañé-Martínez, Jaume Sastre-Garriga, Lluís Ramió-Torrentà, Carmen Bertran-Noguer

**Affiliations:** ^1^Department of Nursing, Health and Health Care Research Group, University of Girona, Girona, Spain; ^2^Department of Neurology, Centre d'Esclerosi Múltiple de Catalunya (Cemcat), Vall d’Hebron Barcelona Hospital Campus, Barcelona, Spain; ^3^Grup d’Investigació Multidisciplinari d’Infermeria, Vall d’Hebron Institut de Recerca (VHIR), Hospital Universitari Vall d’Hebron, Vall d’Hebron Barcelona Hospital Campus, Barcelona, Spain; ^4^Expert Patient Programme Catalonia, Integrated Care Strategy, General Directorate for Health Planning, Department of Health, Generalitat de Catalunya, Barcelona, Spain; ^5^Girona Neuroimmunology and Multiple Sclerosis Unit, Department of Neurology, Dr. Josep Trueta University Hospital and Santa Caterina Hospital, Girona, Spain; ^6^Neurodegeneration and Neuroinflammation Research Group, Girona Biomedical Research Institute (IDIBGI), Salt, Spain; ^7^Statistics and Bioinformatics Unit (UEB), Vall d’Hebron Recerca Institute (VHIR), Vall d’Hebron Barcelona Hospital Campus, Barcelona, Spain; ^8^Department of Genetics, Microbiology and Statistics, University of Barcelona, Barcelona, Spain; ^9^Department of Pharmacy, Vall d’Hebron University Hospital, Barcelona, Spain; ^10^Basic, Translational and Clinical Pharmacy Research Group, Vall d’Hebron Institute of Research (VHIR), Barcelona, Spain; ^11^MS-Neuroimmunology Unit and Laboratory of Advanced Imaging in Neuroimmunological Diseases (ImaginEM), Hospital Clinic Barcelona and Institut d'Investigacions Biomèdiques August Pi i Sunyer (IDIBAPS), University of Barcelona, Barcelona, Spain; ^12^Department of Neurology, Hospital Universitario Arnau de Vilanova, Institut de Recerca Biomèdica de Lleida-IRBLleida, Lleida, Spain; ^13^Neurologist MS Unit, Hospital Germans Trias i Pujol, Badalona, Spain; ^14^Nurse MS Unit, Hospital Germans Trias i Pujol, Badalona, Spain; ^15^Hospital Universitari Sant Joan de Reus, Clinical and Epidemiological Neuroscience Group (NeuroÈpia), Institut d' Investigació Sanitària Pere Virgili (IISPV), Reus, Spain; ^16^Department of Nursing, Centro Neurorehabilitador Mas Sabater, Fundació Esclerosi Múltiple, Reus, Spain; ^17^Department of Neurology, Hospital Universitari de Tarragona Joan XXIII, Tarragona, Spain; ^18^Department of Medical Sciences, University of Girona, Girona, Spain

**Keywords:** multiple sclerosis, expert patient program, nursing-led intervention, disease-related knowledge, quality of life, lifestyle habits, disability status, empowerment

## Abstract

**Background:**

Multiple sclerosis (MS) is associated with complex needs and demands, which require patient-centered care. Expert patient (EP) programs foster knowledge transfer through peer learning, facilitating patients’ empowerment to self-manage their disease. Based on a previous focus group study, we designed an EP program for MS coordinated by nursing professionals for implementation in the different MS reference units of Catalonia (Southwestern Europe). This study aimed to deploy a nurse-led Expert Patient Program of Catalonia™ (EPPC) for people with MS and evaluate its impact on disease-related knowledge, empowerment, and health indicators.

**Methods:**

Pre-test, post-test interventional, multicenter study conducted between January 2021 and December 2023 (NCT NCT04988880). Six MS teams recruited 12 groups of people with relapsing and progressive MS. Participants attended nine virtual sessions led by an EP, trained and supported by a nurse. Questionnaires were delivered after certain sessions and at 6 and 12 months.

**Results:**

Fifty-five participants with relapsing disease and 57 with progressive disease received the intervention. Nine of 18 knowledge questions showed significantly higher post-test vs. pre-test correct answers. Hospital Anxiety and Depression Scale remained unchanged for anxiety and transiently increased for depression, whereas the Patient Activation Measure-13 increased at 12 months, by mean (SD) 2.04 [5.88] points (*p* = 0.0001) in patients with relapsing MS and by 3.28 (5.24) points (*p* = 0.0004) in those with progressive MS. Lifestyle habits remained mostly unchanged, except for medication self-management and diet, whereas visits to nurses and other professionals in MS units significantly decreased. Physical health composite scores in the MS quality of life-54 questionnaire decreased, while the mental health composite scores remained unchanged. Fatigue Severity Scale scores remained unchanged and Expanded Disability Status Scale scores increased in participants with progressive disease. In conclusion, the nurse-led program was successfully implemented across Catalonia and resulted in increased MS knowledge and patient activation, impacting medication self-management, diet, and visits to certain professionals in MS units, despite decreased quality of life and disability in participants with progressive disease.

## 1 Introduction

Multiple sclerosis (MS) is a chronic degenerative disorder of the central nervous system that may lead to physical disability and cognitive impairment, causing a profound impact on quality of life (QoL) (1). Due to the variable disease course, people living with MS face uncertainty regarding the progression of their disease, leading to stress, anxiety, and depression, with a further impact on QoL (2). Like other chronic conditions needing long-term care, MS requires multidisciplinary patient-centered management to meet the specific and complex needs of individuals affected by the disease (3).

One of the goals of the WHO global strategy on integrated people-centered health services is empowering and engaging individuals through the implementation of health education, self-management, peer support, and expert patient groups, among other policies (4). Other key elements of people-centered care are communication between patients and healthcare providers and shared decision-making (SDM) (4, 5), which requires balanced information and patient participation during face-to-face consultations (6). Disease-related knowledge is fundamental for patient empowerment and engagement and is an essential part of the SDM process, led and facilitated by professional nurses (7). In this regard, people with MS should be provided accessible, clear, concise information, as well as empowering education programs (8, 9).

Different strategies for providing MS-related information have been assessed, with varying results on disease-related knowledge and health outcomes (10–12). Nurse-led expert patient (EP) or peer-support programs have been implemented to foster knowledge transfer among individuals with the same chronic condition, aiming to empower patients to self-manage their disease (9, 13, 14). Expert patient programs (EPPs) are peer-learning sessions in small participant groups led by an EP, a non-healthcare professional with the same chronic condition, usually trained by a nurse (13, 15). The EP transfers knowledge and skills in these sessions based on their experience. These programs aim to promote patients’ self-efficacy and self-management, and improve their confidence and resourcefulness (9, 16).

In Catalonia, the Health plan for 2011–2015 and subsequent plans included the Expert Patient Program of Catalonia™ (EPPC™), consisting of a peer-led learning intervention aimed at transferring knowledge, reducing the impact of the disease, and encouraging the acquisition of healthy habits and lifestyles (17, 18). This program has been applied in several chronic conditions, such as cardiovascular and pulmonary disorders, diabetes mellitus, depression, and obesity (19–21), but a specific EPPC for people with MS has yet to be implemented.

Based on a focus group study assessing the needs and demands of people with MS, we designed a specific EPPC for people with MS (EPPC-MS) (15), which was evaluated in a pilot study (to be published elsewhere). This pre-test, post-test, interventional, multicenter study aimed to deploy the nurse-led EPPC-MS across Catalonia and evaluate its impact on disease-related knowledge, empowerment, and health indicators after program completion and in the long term.

## 2 Materials and methods

### 2.1 Study design and population

This pre-test, post-test interventional, multicenter study included patients with progressive and relapsing MS. The study was conducted between January 2021 and December 2023 in seven MS units across Catalonia: the Neuroimmunology and Multiple Sclerosis Unit of Girona (UNIEMTG, Girona), the Vall d’Hebron Hospital Multiple Sclerosis Centre of Catalonia (Cemcat, Barcelona), the MS-Neuroimmunology Unit of Hospital Clínic de Barcelona, the Demyelinating Diseases Unit of the Germans Trias i Pujol University Hospital (Badalona), the Arnau Vilanova University Hospital (Lleida), and, participating as a joint team, the Joan XXIII University Hospital (Tarragona), the Sant Joan University Hospital (Reus). Each unit recruited two groups of people with two forms of MS: one with relapsing MS and one with progressive MS, resulting in a total of 12 groups. Each group participated in an EPPC-MS consisting of nine weekly remote sessions conducted by an EP. Therefore, the groups were formed based on the concept of type of MS (i.e., progressive and relapsing), and study outcomes were analyzed globally and according to MS type, without considering other MS subtypes. The study was conducted in four phases: in the first phase, three groups of participants from three different units underwent the intervention simultaneously. In the next phase, the remaining groups from the same three units underwent the intervention, and so on for the six groups from the three remaining units.

Baseline assessments (pre-test) were performed during the screening visit, before starting the intervention (session 1) or early during the intervention (session 2), and follow-up assessments (post-test) were performed at the end of the intervention (sessions 8 and 9), and at 6 and 12 months after the intervention (Supplementary Table S1). The study protocol was registered on clinicaltrials.gov (Registration number: NCT04988880) and has been published in this journal (22). This study was conducted following the principles of the Declaration of Helsinki and the current local legislation regarding the confidentiality of personal data in clinical studies (Organic Law 3/2018); all participants provided written informed consent. The Clinical Research Ethics Committee of the University Hospital of Girona approved the study protocol (approval code 2020.228; 4 February 2021).

### 2.2 Study population and selection

Nursing personnel involved in patients’ routine care from each participating unit identified eligible patients with MS to join as participants or EPs based on their MS-related knowledge and ability for self-care and self-management, and invited them to participate. Healthcare or educational professionals were ineligible as EPs. Inclusion criteria for participants and EPs were being ≥18 years old and confirmed availability to attend at least 80% of the sessions. Participants were also required to have reported the need for support for disease self-management or to improve MS-related knowledge. EPs were required to have MS-related knowledge and an MS diagnosis before 2018 to ensure sufficient accrual of MS-related knowledge and a long-term relationship with the multidisciplinary team. Eligible EPs were required to have the ability to self-manage and self-care, and a positive perception of the disease. Although no objective instrument or scale was used to assess EP inclusion criteria, professionals from MS units followed patients closely, allowing them to appraise their characteristics and skills.

We excluded patients (as participants and EPs) unable to speak or write in Spanish or Catalan, with aphasia or an auditory disorder preventing them from interacting with the group, with severe cognitive impairment, defined as a score of 1.5-fold the standard deviation below the population mean in one of the three subtests of the Spanish version of the Brief International Cognitive Assessment for Multiple Sclerosis (BICAMS) (23, 24), with severe emotional impairment, defined as a score above 11/21 on any of the two Hospital Anxiety and Depression Scale (HADS) subscales (anxiety and depression) (25), and with any psychopathological comorbidity or mental disorder diagnosis that may restrict their interaction with the group.

The participating units selected three EP candidates each. The principal investigator and the institutional managers of the EPPC further assessed their adequacy during remote interviews and selected one EP with relapsing MS and one with progressive MS for each participating unit. The selected EPs were provided with the session materials and trained for their role to expand their knowledge of the sessions’ contents and gain confidence to lead and answer any questions from the participants.

### 2.3 Intervention

Participants attended nine 90-min weekly sessions led by an EP, during which they shared their knowledge and experience on all aspects of MS and self-management. The sessions were conducted remotely on an online platform, which sent reminders before each session. The content of the sessions are described in Supplementary Table S2 and were identified in a previous pivotal study (15). Two nurses ─the study’s PI and one member of the EPPC panel of directors’─ acted as observers in all sessions, provided help and support to the EP if needed, and assessed each session jointly with the EP at the end.

The sessions were divided into two blocks: a 30-min theoretical introduction conducted by the EP to focus on the day’s topic and a practical 60-min block, during which the EP encouraged interaction to facilitate the communication of doubts, questions, or experiences related to the context, which were solved and discussed collectively within the group. Patients were grouped based on the type of MS to facilitate the sharing of experiences within homogenous groups and support the leadership of the EP. This concept adhered to the recommendations of the target population, as identified in our previous focus group study (15).

### 2.4 Outcomes and variables

The primary objective of this study was the implementation of the EPPC-MS across Catalonia, according to the Catalan Health Plan for 2016–2020 and the current Health Plan (18, 26). Secondary objectives were to assess the effectiveness of the program in improving disease-related knowledge, the impact on patients’ emotional status, habits and lifestyle, engagement, and health indicators, and satisfaction with the program.

Knowledge of MS was assessed using a specific questionnaire developed by the expert panel of the EPPC, including neurologists, nurses, psychologists, neuropsychologists, occupational therapists, physiotherapists, primary care physicians, and experts in health literacy, based on a previous pilot qualitative study and on patients’ perspectives (15). The questionnaire comprised 18 questions with four possible answers, and the results were analyzed as a categorical variable (distribution of answers) (Supplementary Table S3). The questionnaire was administered before session 1, after session 8, and at 6 and 12 months after the intervention.

The impact of the intervention on emotional status was assessed using the validated Catalan and Spanish versions of the HADS, a 14-item questionnaire comprising the anxiety and depression subscales (HADS-anx and HADS-dep, respectively), with seven items each (27, 28). Scores range from 0 to 3, from minimum to maximum affectation. The questionnaire was administered at the screening visit, after session 8, and 6 and 12 months after the intervention.

Patients’ engagement was assessed using the validated Spanish version of the Patient Activation Measure-13 (PAM-13) questionnaire (29), comprising 13 items rated on a 1–4 scale, from 1: “strongly agree” to 4: “strongly disagree.” The PAM items are framed within four domains, including knowledge, beliefs, confidence, and skills on managing one’s health. To calculate the PAM-13 score, the sum of all scores is divided by the number of answers and multiplied by 13, and the result is further transformed to a theoretical 0–100-point scale, based on a calibrated scale range from 38.6 to 53.0. Higher scores indicated increased activation (30). It was administered at the beginning of the intervention (session 2), after session 9, and at 6 and 12 months after the intervention. To assess the impact of the program on patients’ habits and lifestyles, we used an MS-specific questionnaire developed by the EPPC, with 12 questions and 2–5 possible answers. The results were analyzed as a categorical variable (distribution of answers to each question). Even though the questionnaire is awaiting validation, similar questionnaires have been used to assess other variants of the EPPC. Healthcare service utilization by patients, including visits to the MS units, primary care, and emergency room, was used as an indirect measure of patients’ empowerment in disease self-management. Similarly, compliance with treatment was used as a surrogate measure of activation and was measured as the percentage of retrieved medications of those prescribed. Data were collected one year before and after the intervention from regional health and pharmacy registries.

Health indicators included fatigue management, disability, and QoL. Fatigue was assessed using the validated Spanish version of the Fatigue Severity Scale (FSS) (31), a 9-item self-administered questionnaire rated on a 1–7 scale, with 1 being “strongly agree” and 7 “strongly disagree.” FSS was assessed after session 2, session 9, and at 6 and 12 months. Disease evolution was also considered and was assessed by neurologists at the screening visit and 12 months after the end of the intervention using the Expanded Disability Status Scale (32), with scores ranging from 0 (normal neurological status) to 10 (death due to MS). We assessed QoL using the Spanish-validated version of the widely used MSQoL-54 questionnaire (33), a 54-item self-administered questionnaire with 36 general QoL questions and 18 MS-specific questions, which generate 12 subscales, two composite summary scores corresponding to mental and physical health, and two individual questions measuring changes in health status and satisfaction with sexual function. Scores range from 0 to 100, with higher scores indicating better QoL.

Participants’ and EPs’ satisfaction with the program was also assessed immediately after the intervention (session 9) and 12 months after the intervention. We used specific 13- and 6-item questionnaires for participants and EPs on a 5-point Likert scale from 0 (not at all satisfied/agree) to 5 (very much satisfied/agree). The questionnaires were developed by the EPPC expert panel and were used for other program variants (20, 34).

### 2.5 Statistical analysis

The sample size was estimated based on the deployment of the EPPC-MS across Catalonia (primary outcome). It was calculated by prioritizing those units willing to participate in the study and interested in implementing the program in the future to offer it to the people with MS who were regularly followed up on at the unit. Each participating unit included one group with relapsing MS and one with progressive MS, with a target of 12 participants per group (144 in total) based on the methodology used in the program. The group size was expected to compensate for potential losses and ensure a minimum of 8–10 participants per group.

Categorical variables were described as frequencies and percentages, and quantitative variables with the mean and standard deviation (SD) or the median and interquartile range (IQR: Q1, Q3). Patient groups according to MS type were compared using the Chi-square or Fisher’s tests for categorical variables and U Mann–Whitney tests for quantitative variables. Changes in study variables throughout time were analyzed using the Skillings-Mack test for categorical variables and a repeated-measures analysis of variance (ANOVA) for quantitative variables. Changes were assessed for the total of participants and according to MS, except for disease-related knowledge, lifestyle and habits, and treatment compliance. Statistical significance was set at a two-sided alpha <0.05 for all analyses. All statistical analyses were performed using Stata v.18.0 (RRID:SCR_012763).

## 3 Results

### 3.1 Demographic and clinical characteristics of study patients

**Figure 1:**
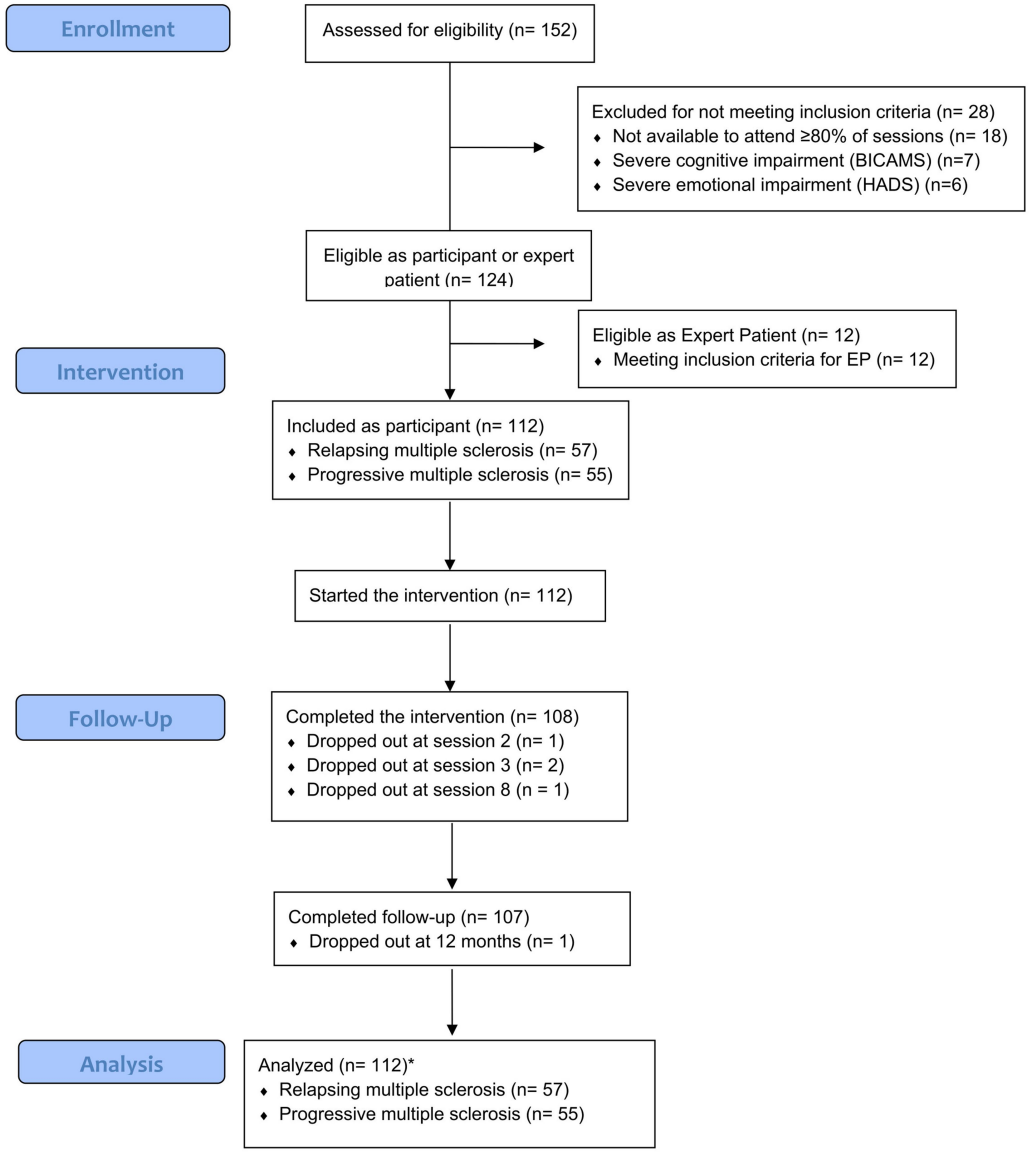
Flow chart of study participants. BICAMS, Brief International Cognitive Assessment for Multiple Sclerosis; EP, expert patient; HADS, Hospital Anxiety and Depression Scale.

We invited 8 MS units in Catalonia, of which 7 accepted participation, and recruited 152 patients with MS, 79 with relapsing MS, and 73 with progressive MS as participants and EPs. Of these, 28 failed to meet the inclusion criteria, and 12 met the inclusion criteria for EPs. The remaining 112 patients, 55 with relapsing MS and 57 with progressive MS, were included as participants; most completed the intervention (*n* = 108, 96.4%) (Figure 1). The number of participants per group ranged from 7 to 12, and their distribution according to type of MS and participating unit are summarized in Supplementary Table S4.

Participants were mostly female, particularly those with relapsing MS (*p* = 0.0055), and were older in the progressive MS group (*p* = 0.0001) (Table 1). Participants with progressive MS had higher EDSS scores and were retired or were granted a disability pension, and received disease-modifying treatment (DMT) administered at the hospital more frequently. The sociodemographic and clinical characteristics of expert patients were similar to those of participants (Supplementary Table S5).

**Table 1:** Baseline sociodemographic and clinical characteristics of participants according to multiple sclerosis type, *N* = 112.

	Relapsing MS *n* = 57	Progressive MS *n* = 55	*p*-value
Sociodemographic characteristics			
Sex, *n (%)*			
Female	46 (80.7)	31 (56.4)	0.0055^a^
Male	11 (19.3)	24 (43.6)	
Age (years), *median (IQR)*	44.0 (38.0, 50.0)	50.0 (46.0, 58.0)	0.0001^b^
Marital status, *n (%)*			
Single, separated, divorced, widow(er)	12 (21.8)	24 (21.4)	0.9214^a^
Married, with partner	43 (78.2)	88 (78.6)	
Support from relatives, *n (%)*			
Yes	56 (98.2)	54 (98.2)	1.0000^c^
No	1 (1.8)	1 (1.8)	
Educational level, *n (%)*			
Primary school	10 (17.5)	11 (20.0)	0.9033^a^
High school and professional degree	22 (38.6)	22 (40.0)	
Advanced studies (university, master’s, and doctorate)	25 (43.9)	22 (40.0)	
Employment			
Employed	44 (77.2)	13 (23.6)	<0.0001^a^
Retired, disability pension	13 (22.8)	42 (76.4)	
Clinical and treatment characteristics			
EDSS scores, *median (IQR)*	2.00 (1.50, 3.00)	6.00 (5.00, 6.50)	0.0000^b^
Family history of MS, *n (%)*			
Yes	2 (3.5)	2 (3.6)	1.0000^c^
No	55 (96.5)	53 (96.4)	
Disease-modifying treatment, *n (%)*			
Yes	49 (86.0)	36 (65.5)	0.0112^a^
No	8 (14.0)	19 (34.5)	
Type of disease-modifying treatment, *n (%)*	*n* = 49	*n* = 36	
Self-administered	29 (59.2)	3 (8.3)	<0.0001^a^
Hospital administered	20 (40.8)	33 (91.7)	

EDSS, Expanded Disability Status Scale; IQR, interquartile range; MS, multiple sclerosis; SD, standard deviation.

a Chi-squared test.

b U Mann–Whitney test.

c Fisher’s exact test.

### 3.2 Changes in disease-related knowledge

Answers to nine of the 18 questions in the knowledge questionnaire significantly changed, with higher rates of correct answers at session 8 and thereafter compared to session 1 (Table 2), indicating improved and maintained knowledge 12 months after the intervention. These questions were related to the characteristics of relapsing–remitting MS, MS diagnosis, diet, weight, disease appearance, daily life activities, urinary and fecal incontinence, and sexual function. The remaining nine questions, except the one related to McDonald criteria, were answered correctly by most patients (>⁓80%) at session 1, and some of them tended to increase rates of correct answers at session 8, although differences lacked statistical significance (Supplementary Table S6).

**Table 2:** Patients’ answers to the 18-question knowledge test at the indicated timepoints (only questions with statistically significant differences are shown), *N* (%).

	S1	S8	6 m	12 m	*p-*value^1^
**Q2. The relapsing–remitting form of MS:**	***N* = 112**	***N* = 108**	***N* = 101**	***N* = 106**	0.001
(a) Is the most frequent.	18 (16.07)	13 (12.04)	10 (9.9)	11 (10.38)	
(b) Is characterized by the presence of flares.	17 (15.18)	8 (7.41)	8 (7.92)	9 (8.49)	
(c) The symptoms of a flare usually develop over hours or days.	1 (0.89)	1 (0.93)	0 (0)	0 (0)	
(d) All of the above are correct.	76 (67.86)	86 (79.63)	83 (82.18)	86 (81.13)	
**Q3. Currently, a MS diagnosis:**	***N* = 112**	***N* = 108**	***N* = 101**	***N* = 106**	<0.0001
(a) Can only be established by MRI findings.	37 (33.04)	16 (14.81)	11 (10.89)	11 (10.38)	
(b) Can only be established by cerebrospinal fluid findings.	11 (9.82)	4 (3.7)	2 (1.98)	2 (1.89)	
(c) Can only be established through neurological exploration.	1 (0.89)	1 (0.93)	0 (0)	0 (0)	
(d) Is based on the McDonald diagnostic criteria.	63 (56.25)	87 (80.56)	88 (87.13)	93 (87.74)	
**Q6. Diet and MS**	***N* = 112**	***N* = 108**	***N* = 100**	***N* = 106**	<0.0001
(a) No relationship exists between diet and MS.	21 (18.75)	5 (4.63)	8 (8.0)	9 (8.49)	
(b) A diet high in salt, fat, and red meat is recommended.	1 (0.89)	0 (0)	1 (1.0)	0 (0)	
(c) The Mediterranean diet is the recommended diet.	87 (77.68)	103 (95.37)	89 (89.0)	95 (89.62)	
(d) Gluten should be eliminated and carbohydrates reduced.	3 (2.68)	0 (0)	2 (2.0)	2 (1.89)	
**Q10. Weight and MS:**	***N* = 112**	***N* = 108**	***N* = 101**	***N* = 106**	0.046
(a) Being overweight worsens the progression of the disease.	2 (1.79)	1 (0.93)	2 (1.98)	0 (0)	
(b) Keeping an adequate weight is recommended, since it helps carry out the activities of daily living.	13 (11.61)	8 (7.41)	3 (2.97)	5 (4.72)	
(c) Regular exercise helps maintain an adequate weight.	0 (0)	0 (0)	0 (0)	2 (1.89)	
(d) All of the above are correct.	97 (86.61)	99 (91.67)	96 (95.05)	99 (93.4)	
**Q11. MS and daily life abilities:**	***N* = 112**	***N* = 108**	***N* = 101**	***N* = 106**	0.007
(a) It is crucial to know and value one’s abilities.	10 (8.93)	3 (2.78)	3 (2.97)	3 (2.83)	
(b) Abilities must be used wisely.	0 (0)	1 (0.93)	1 (0.99)	0 (0)	
(c) You must value and love yourself.	4 (3.57)	1 (0.93)	1 (0.99)	1 (0.94)	
(d) All of the above are correct.	98 (87.5)	103 (95.37)	96 (95.05)	102 (96.23)	
**Q12. When MS appears:**	***N* = 112**	***N* = 108**	***N* = 101**	***N* = 106**	0.038
(a) We do not accept the disease; we reject it.	24 (21.43)	18 (16.67)	16 (15.84)	12 (11.32)	
(b) It is a travel companion for life.	88 (78.57)	90 (83.33)	85 (84.16)	94 (88.68)	
(c) It affects everyone in a similar way.	0 (0)	0 (0)	0 (0)	0 (0)	
(d) The disease lasts a few months.	0 (0)	0 (0)	0 (0)	0 (0)	
**Q16. Urinary incontinence:**	***N* = 112**	***N* = 108**	***N* = 101**	***N* = 106**	<0.0001
(a) When there is an urgency to urinate, an increase in frequency and/or a feeling of not emptying the bladder properly, you should consult with the healthcare team.	45 (40.18)	26 (24.07)	20 (19.8)	21 (19.81)	
(b) Urinary tract infections can increase fatigue and spasticity.	5 (4.46)	1 (0.93)	2 (1.98)	1 (0.94)	
(c) Bladder catheterization reduces the frequency of infections.	0 (0)	1 (0.93)	0 (0)	0 (0)	
(d) All of the above are correct.	62 (55.36)	80 (74.07)	79 (78.22)	84 (79.25)	
**Q17. Fecal incontinence:**	***N* = 112**	***N* = 108**	***N* = 101**	***N* = 106**	<0.0001
(a) Constipation is the most common intestinal disorder in people with MS.	4 (3.57)	1 (0.93)	2 (1.98)	7 (6.6)	
(b) The Mediterranean diet, drinking plenty of water and exercising can help avoid constipation.	26 (23.21)	10 (9.26)	11 (10.89)	10 (9.43)	
(c) Laxatives must be taken according to the instructions of professionals.	3 (2.68)	1 (0.93)	1 (0.99)	0 (0)	
(d) All of the above are correct.	79 (70.54)	96 (88.89)	87 (86.14)	89 (83.96)	
**Q18. MS and sexual function**	***N* = 112**	***N* = 108**	***N* = 101**	***N* = 106**	0.001
(a) Sexual dysfunction may be related with the injuries and symptoms caused by the disease, and with psychological, educational, and sociocultural factors.	5 (4.46)	4 (3.7)	1 (0.99)	2 (1.89)	
(b) Some treatments, such as antidepressants, can cause sexual dysfunction.	2 (1.79)	0 (0)	0 (0)	0 (0)	
(c) Sexual dysfunction must be discussed openly within the couple and the treatment must be multidisciplinary.	9 (8.04)	2 (1.85)	3 (2.97)	3 (2.83)	
(d) All of the above are correct.	96 (85.71)	102 (94.44)	97 (96.04)	101 (95.28)	

6 m, 6 months; 12 m, 12 months; MS, multiple sclerosis; SX, Session X. *p*-values were calculated using the Skillings-Mack test.

### 3.3 Emotional impact

**Figure 2:**
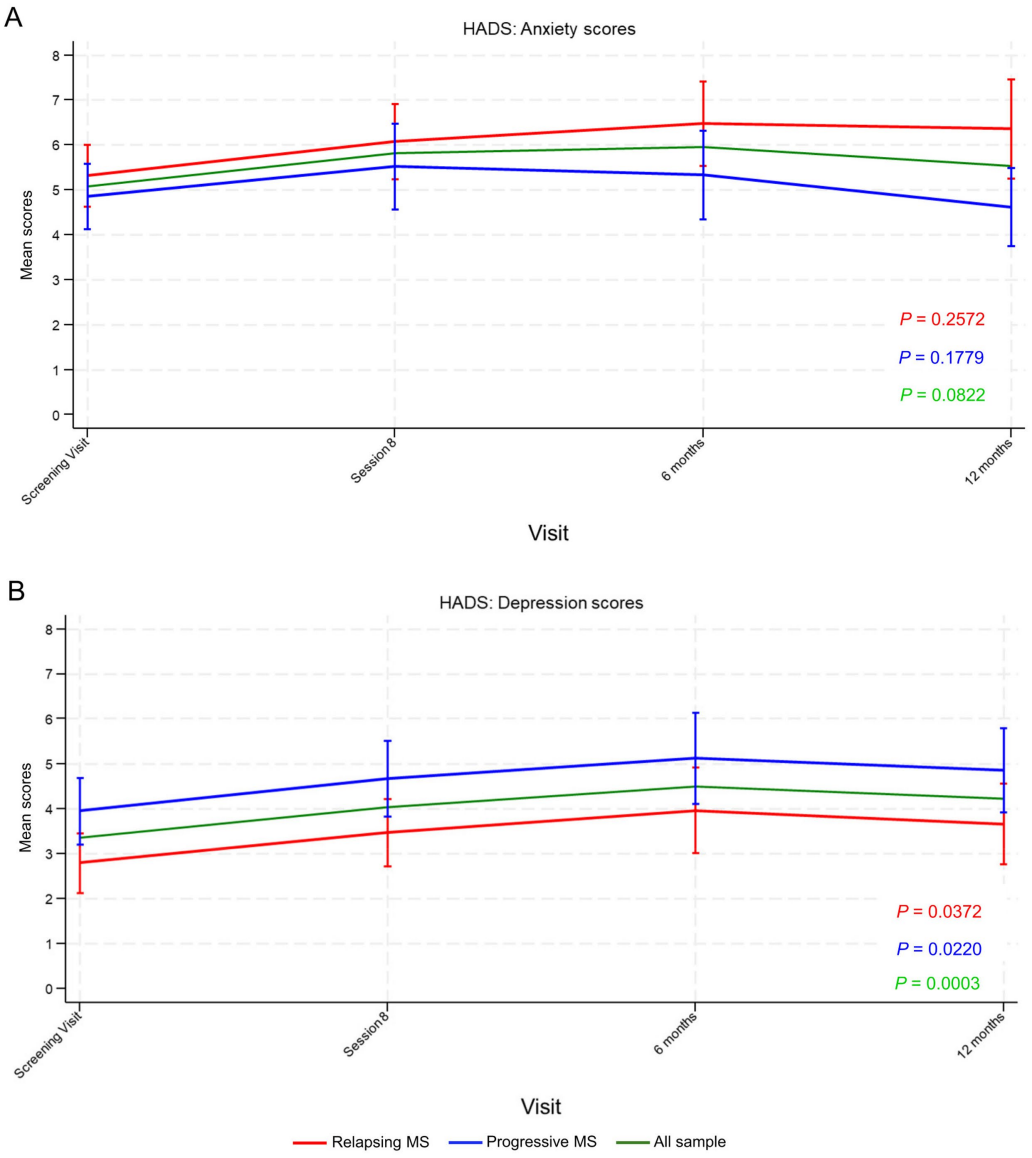
Hospital Anxiety and Depression Scale (HADS) **(A)** anxiety and **(B)** depression scores from the screening visit until 12 months’ follow-up. Data points represent mean scores and vertical lines the 95% confidence interval (CI). *p*-values were calculated using the repeated measures analysis of variance (ANOVA). MS, multiple sclerosis.

HADS-anxiety scores lacked statistically significant variations from baseline throughout study visits, although they appeared to follow opposite patterns in the relapsing and progressive MS groups (Figure 2A). In the relapsing group, scores appeared to increase at the 6-month follow-up (indicating a trend toward worsened anxiety) and subsequently plateaued. In contrast, in the progressive group, scores appeared to remain constant at 6 months and subsequently decreased at 12 months (indicating a trend toward improved anxiety). In contrast, the mean HADS-depression score significantly increased globally and according to MS type, indicating increased depression, particularly between the screening visit and the 6-month follow-up, with a slight decrease at 12 months (Figure 2B).

### 3.4 Impact on participants’ engagement and lifestyle and habits

**Figure 3:**
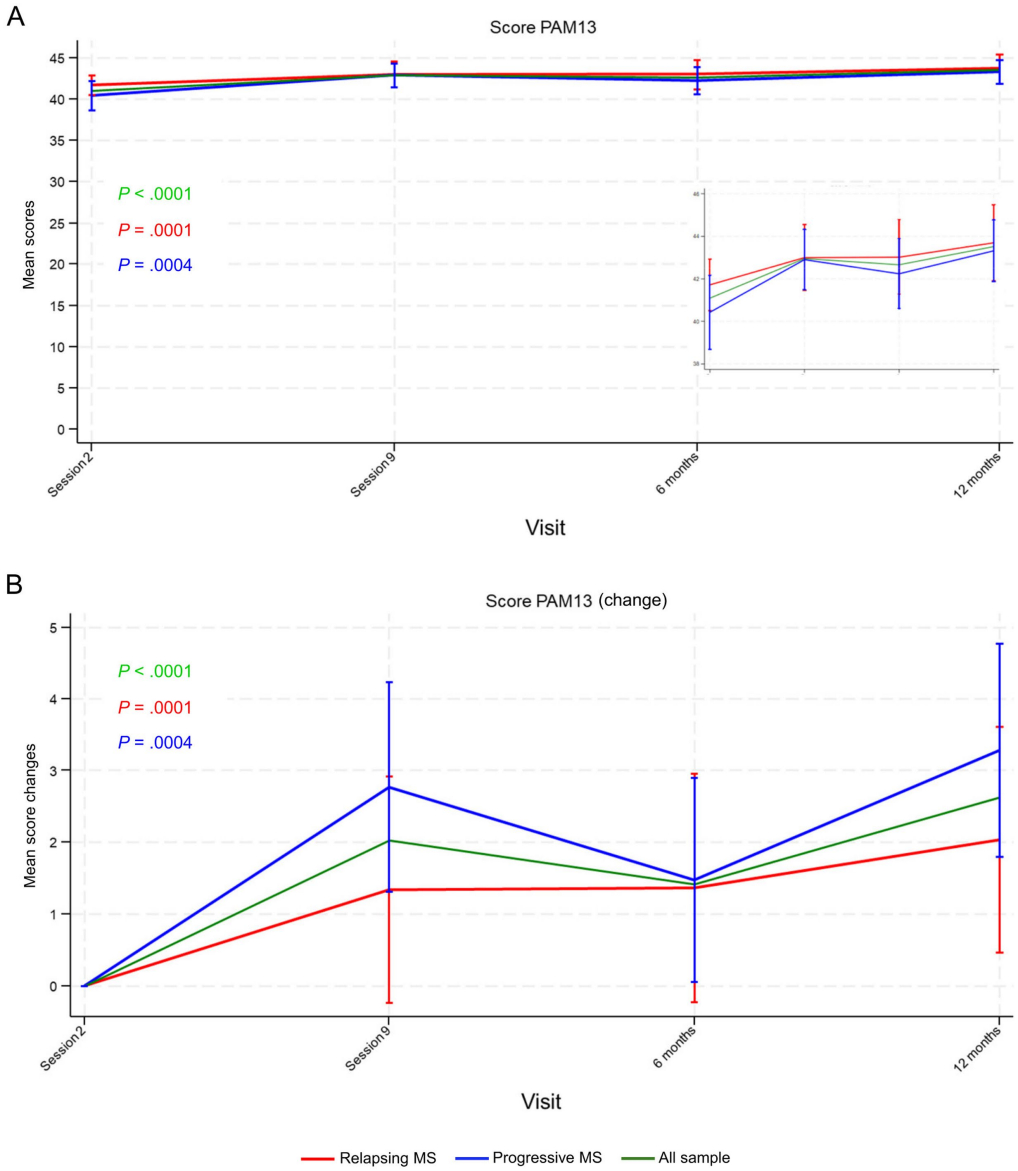
Patient Activation Measure-13 (PAM-13) **(A)** mean scores and **(B)** mean score changes from session 2 until 12 months’ follow-up. The inset in **(A)** shows a close-up with the scale set between 38 and 46 points. Data points represent means (scores in **A** and score changes in **B**) and vertical lines the 95% confidence interval (CI). *p*-values were calculated using the repeated measures analysis of variance (ANOVA). MS, multiple sclerosis.

Mean PAM-13 scores, a measure of participants’ engagement, were slightly higher in participants with relapsing MS (41.72 [SD = 4.53]) than in those with progressive MS (40.43 [SD = 6.42]) before the intervention. Scores significantly increased from session 2 and globally throughout the study (mean [SD] increase of 2.62 [5.60] points at 12 months, *p* < 0.0001), in participants with relapsing MS (mean [SD] increase of 2.04 [5.88] points at 12 months, *p* = 0.0001), and in those with progressive MS (mean [SD] increase of 3.28 [5.24] points at 12 months, *p* = 0.0004) (Figure 3). Scores increased between session 2 and session 9, with a further increase during follow-up (between 6 and 12 months), indicating persistent or increased activation after program completion (Figure 3).

Most participants’ habits and lifestyle items remained unchanged throughout the study, except those regarding the site of medication administration, missing medication doses, self-medication, other physical activities carried out, and kind of diet (Supplementary Table S7). Thus, after the intervention and during follow-up, patients reported increased frequency of medication administration at the hospital (vs. at home), decreased missed medication doses, self-medication, and decreased physical activity in the “other” category. Moreover, although the number of participants on a diet did not change, more participants followed fat-free and low-sugar diets, while the frequency of diets in the category “other” decreased.

Treatment compliance remained unchanged at 12 months with respect to the screening visit (*p* = 0.9605), with mean (SD) compliance of 98.80 (3.77) % at screening (*n* = 82) and 99.0 (3.04) % at 12 months (*n* = 80).

**Figure 4:**
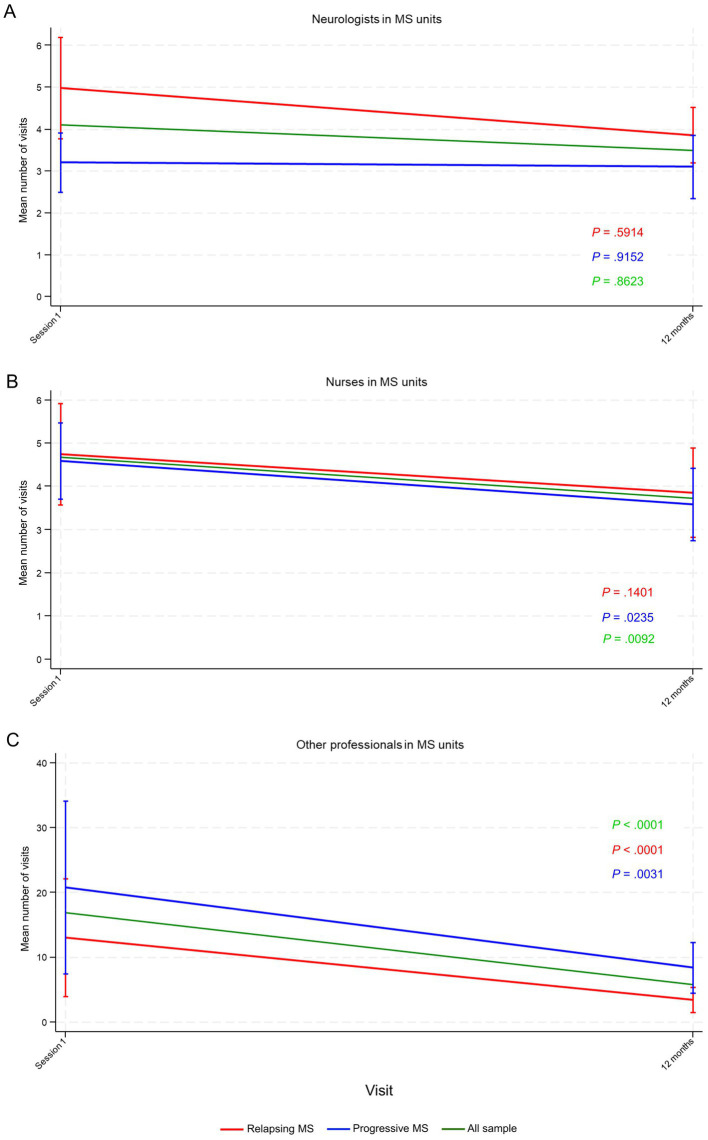
Number of visits to healthcare professionals in multiple sclerosis (MS) units during the previous year before and after the intervention, including **(A)** neurologist, **(B)** nurses, and **(C)** other professionals. Data points represent mean number of visits and vertical lines the 95% confidence interval (CI). *p*-values were calculated using the repeated measures analysis of variance (ANOVA).

The number of visits to emergency care during the previous year before and after receiving the intervention remained unchanged (Supplementary Table S8). Similarly, the number of visits to MS units remained stable overall regardless of visit type. However, visits to nurses significantly decreased in all participants (*p* = 0.0092) and those with progressive MS (*p* = 0.0235), albeit modestly (Figure 4 and Supplementary Table S8). Visits to other professionals in MS units showed a marked significant decrease for all participants (*p* < 0.00001) and for those with relapsing (*p* < 0.00001) and progressive MS (*p* = 0.0031), whereas visits to neurologists in MS units remained unchanged (Figure 4 and Supplementary Table S8).

### 3.5 Changes in health indicators

**Figure 5:**
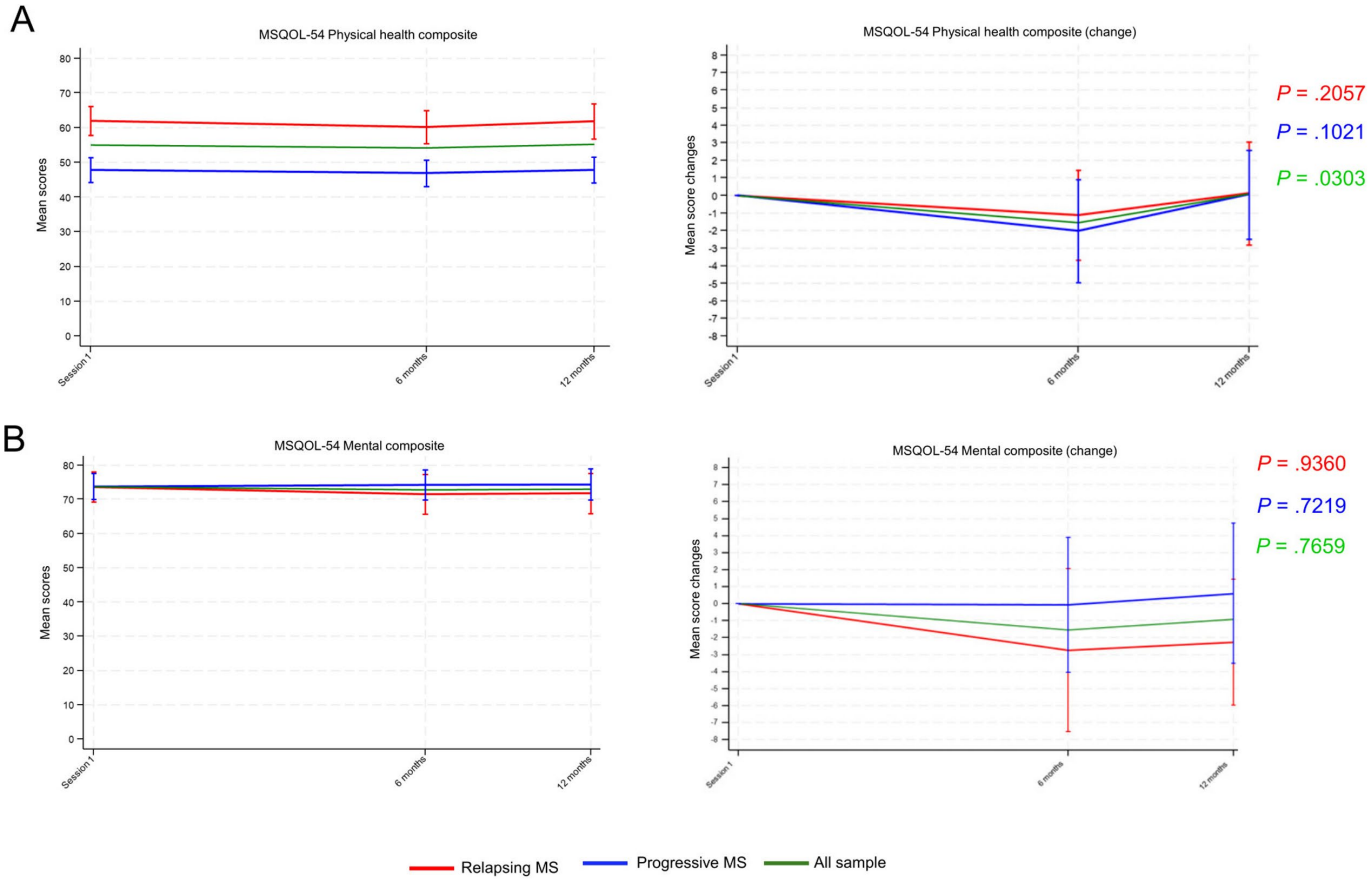
Multiple Sclerosis Quality of Life-54 mean scores for **(A)** Physical health summary composite and **(B)** Mental health summary composite from session 1 until 12 months’ follow-up. Mean scores and mean changes from session 1 are shown in the adjacent graphs. Data points represent means and vertical lines the 95% confidence interval (CI). *p*-values were calculated using the repeated measures analysis of variance (ANOVA). MS, multiple sclerosis.

Regarding the impact of the intervention on QoL, the physical health summary composite scores showed significant changes in all participants (*p* = 0.0303), with an initial decrease at 6 months, but with scores recovering at 12 months (Figure 5A). In contrast, the mental health summary composite scores remained unchanged throughout the study (Figure 5B).

**Figure 6:**
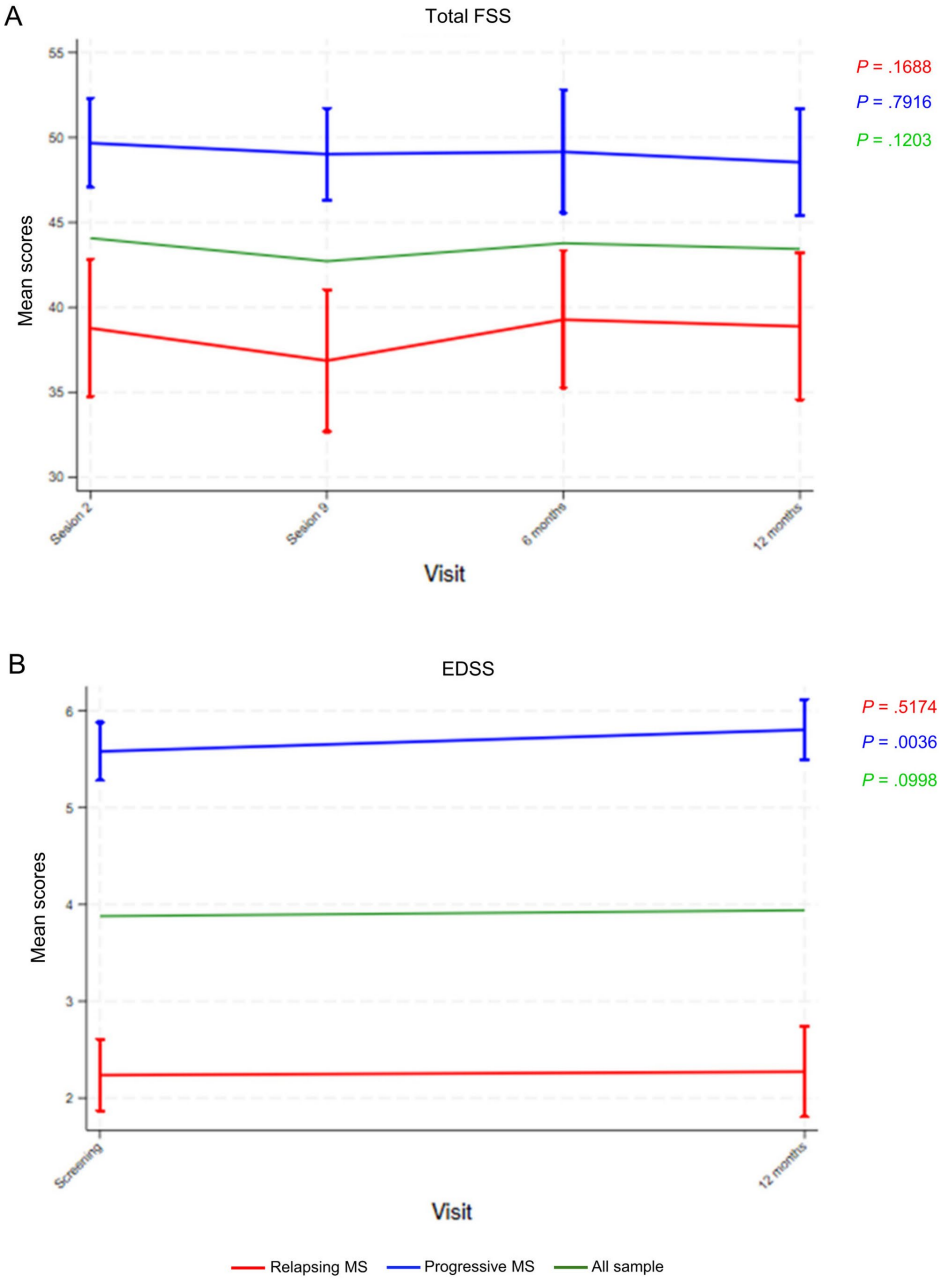
**(A)** Fatigue Severity Scale (FSS) and **(B)** Expanded Disability Status Scale (EDSS) mean scores throughout the indicated study visits. Data points represent means and vertical lines the 95% confidence interval (CI). *p*-values were calculated using the repeated measures analysis of variance (ANOVA). MS, multiple sclerosis.

Total scores for the FSS remained stable in all participants and according to MS group throughout the study (Figure 6A). In contrast, scores for the EDSS significantly increased for participants with progressive MS (*p* = 0.0036) while remaining stable for all participants and those with relapsing MS (Figure 6B).

### 3.6 Participants and expert patients satisfaction with the program

Overall, at the end of the intervention (session 9), most participants agreed or very much agreed with the questions assessing their experience, indicating a positive experience, and their perception remained unchanged during follow-up (12 months) (Supplementary Table S9). Likewise, all 12 EPs agreed or very much agreed that their experience with the program was positive, the information provided by the healthcare professional was clear, the information and supporting documents were appropriate, and the relationship established between the EP and the group was good. All of them very much agreed that they would repeat the experience (Supplementary Table S10). However, EPs’ perceptions on whether leading a group for the first time was complicated were more diverse, with 8 (66.67%) EPs giving a 2–3 score. EPs’ perceptions remained unchanged at 12 months after completing the intervention.

## 4 Discussion

In this pre-test, post-test interventional study including patients with progressive and relapsing MS, we showed that the EPPC-MS was successfully implemented in seven MS units in Catalonia. The peer-led educational intervention for MS patients increased disease-related knowledge and had a positive impact on patient activation, which was maintained during follow-up. Increased knowledge and activation impacted medication-related habits and diet, and decreased the use of healthcare resources, with significantly decreased number of visits, particularly to nurses and other healthcare professionals in MS units. The intervention had no impact on anxiety, although it resulted in a transient increase in depression. Regarding QoL, the physical health composite transiently decreased, and the mental health composite remained unchanged in all participants. Even though disability worsened in participants with progressive MS, fatigue remained unchanged. Participants and EPs reported a positive experience with the intervention.

Previous studies assessing the effectiveness of educational programs for patients with MS on disease-related knowledge are scarce. Moreover, the interventions were highly heterogenous in terms of the strategies used, and some were limited regarding the aspects of MS management covered (10, 35–37). Nevertheless, they increased patients’ knowledge of some MS-related aspects (10, 35–37). Studies assessing other information-sharing strategies have also shown increased disease-related knowledge (12). In this study, the EPPC-MS content covered multiple aspects of MS management, which patients identified as important in a previous nurse-led focus groups study assessing their needs and demands (15). Moreover, given the lack of an MS knowledge questionnaire validated in Spanish and/or Catalan, we developed a tailored questionnaire to determine the impact on knowledge, which we used in a previous pilot study (to be published elsewhere). Overall, answers to the knowledge questionnaire reflected increased MS-related knowledge at the end of the intervention, as in the pilot study, with a similar distribution of answers during follow-up, indicating that knowledge persisted. However, the answers to some questions remained unchanged, reflecting no increase in knowledge regarding some aspects of MS. Although knowledge was already high before the intervention for some questions, the lack of changes in certain questions provided valuable information to improve program contents.

In this study, the peer-led intervention resulted in increased participant activation regardless of MS type. Patient activation has been defined as a process developed in four steps, ranging from patients being relatively passive and not seeing themselves as playing an active role in their health to patients having the knowledge and confidence to self-manage health behaviors and gather additional support when needed (38). Basic knowledge about one’s condition and treatment appears to be important early in this progressive model (38). Patient activation is challenging and, at the same time, it is particularly important in MS owing to its progressive and fluctuating nature and unpredictable relapses (39). Moreover, the adverse events associated with DMTs and the variable effectiveness observed preclude treatment adherence and, thus, engaging MS patients in self-care is particularly relevant (35, 39). In our study, the disease-related knowledge gained after the intervention led to participant activation regardless of MS type, despite the lower PAM-13 scores of participants with progressive MS before the intervention, similarly to previous studies (39). In this regard, mean PAM-13 scores in this study population (41.72, SD = 4.53 for relapsing and 40.43, SD = 6.42 for progressive MS) were lower than those reported in previous studies (64.31, SD = 11.93 for relapsing and 57.39, SD = 10.54 for progressive MS) (39). Furthermore, participants’ activation increased despite the concurrent worsening of disability and depression, the latter being identified as a barrier to patient activation (39, 40). Although the mean increase in activation was relatively modest (2.62 [SD:5.60] points in all participants), previous studies have shown that 1 to 10-point changes may have a significant impact on clinical outcomes in other chronic conditions (41, 42). Moreover, activation is associated with the ability to navigate the healthcare system and, in our study, the intervention resulted in decreased visits to nurses and other healthcare professionals in MS units, possibly reflecting patient activation and empowerment resulting from increased MS knowledge.

Despite the effectiveness of the intervention in improving MS-related knowledge and patient activation, the impact on health-related behaviors and outcomes was modest and mostly limited to medication-related behaviors. While previous studies assessing educational programs and information provision in patients with MS have not investigated the impact on health behaviors (10, 12), increased activation has been shown to improve self-management behaviors in people with chronic conditions (43). Similarly, increased engagement has been associated with healthy lifestyle behaviors in people with MS (11). In this study, the intervention changed lifestyle behaviors related to medication and diet, suggesting a healthier lifestyle and improved treatment adherence. These changes may result from increased patient activation and are very relevant in the management of chronic conditions such as MS, with associated complex needs and uncertain progression.

Regarding QoL, the mental health composite remained unchanged whereas the physical health composite scores decreased transiently, which indicated transiently worsened physical health. This transient change likely reflected disease progression and was not a consequence of the intervention. Previous educational and information provision interventions have shown no or very small effects on QoL (10, 12, 44). Despite the worsened summary physical health composite scores, anxiety and depression remained quite low during follow up and consistently below the cut-off for pathological symptoms. These observations suggest that the increased disease-related knowledge and activation resulting from the intervention may have contained the emotional impact of MS. In line with overall health status, disability increased in participants with progressive MS, whereas fatigue remained constant. Overall, these results are similar to those from lay-led educational programs for people with chronic diseases, which have been shown to increase patients’ confidence to change behavior (i.e., self-efficacy), with no effects on QoL, use of healthcare resources, and other health outcomes (45). Given the progressive and unpredictable features of MS, attaining an impact on health indicators might prove even more challenging. In our study, disability worsened in patients with progressive MS but did not generate demands resulting in an additional burden to MS units. Contrarily, despite the increased disability, visits to certain professionals in MS units decreased and fatigue remained unchanged, suggesting improved fatigue management.

This study successfully implemented the EPPC-MS in seven MS units across Catalonia as part of the strategic goals of the current Health Plan of the Government of Catalonia (18, 26). After completion of the intervention, the final versions of the educational materials and methodological guide of the EPPC-MS were developed jointly and dynamically with contributions from participants, EPs, local investigators, and the MS multidisciplinary team in each center. The EPPC-MS is offered in the seven participating units and this initial implementation provided sufficient expertise to the EPPC-MS coordinating team to transfer the program to other MS units in Catalonia and train them for its successful implementation. Furthermore, participants may be selected as EPs after completing the program in subsequent editions.

The results from this study should be interpreted considering the limitations associated with its pre-test, post-test design, which lacked a control group receiving standard care. However, this study was performed within the EPPC and Health plan frameworks and aimed to deploy the newly developed EPPC-MS; therefore, all patients were offered participation. Other limitations were associated with the outcomes assessed and the instruments used. Given the already high number of questionnaires administered, self-efficacy remained unassessed despite being a relevant outcome associated with disease-related knowledge, activation, and empowerment. However, increasing the number of questionnaires may have resulted in study dropouts. Regarding the questionnaires administered, the MS knowledge and lifestyle habits questionnaires are not standard instruments, precluding comparisons with other studies and awaiting validation (46). Moreover, outcomes were analyzed globally and according to MS type, and the effectiveness of the program in other MS subtypes remained unaddressed. Likewise, this comprehensive study included a large number of variables and detailed analyses were unfeasible, such as the evaluation of changes in the 12 MS-QoL subscales. Importantly, this study design considered the COVID-19 pandemic situation, and included risk mitigation strategies, such as the remote delivery of the intervention. In this regard, the pandemic situation likely influenced in-person visits with healthcare professionals and may have altered the intervention effects on healthcare resource use. However, given the successive COVID-19 waves experienced in Catalonia throughout the study, we believe that the impact of the pandemic on the use of healthcare resources was constant and therefore similar before and after the implementation of the program. Despite these limitations, this study met the primary goal of implementing the EPPC-MS across Catalonia according to the current local Health Plan and showed that participation in the newly designed EPPC-MS resulted in increased MS knowledge and promoted patient activation. Moreover, the experience and satisfaction with the program was positive for participants and EPs. Future studies may focus on qualitatively assessing participants’ and expert patients’ experiences.

## 5 Conclusion

This study reports the successful implementation of a newly developed EPPC for people with MS across Catalonia. Participation in the program resulted in increased knowledge about MS and patient activation, leading to lifestyle changes associated with self-management of medication and diet, and decreased visits to certain healthcare professionals in MS units. Participants and EPs rated participation in the program as a positive experience. The expertise acquired during the initial implementation of the program helped develop the final educational materials and facilitated the implementation of the program in other MS units within the current Health Plan framework.

## Data Availability

The original contributions presented in the study are included in the article/Supplementary material, further inquiries can be directed to the corresponding author.
