# Supplementary material for: An expert patient program for people living with multiple sclerosis improves knowledge and empowerment: a pre-test, post-test multicenter implementation study
Source: Front Neurol. 2025 Apr 29;16:1581038. doi: 10.3389/fneur.2025.1581038 (PMC12071084; doi:10.3389/fneur.2025.1581038)
Supplement: Supplementary file 1 [file Supplementary_file_1.docx]

**Supplementary Materials for the Manuscript “An Expert Patient Program to Improve Empowerment and Quality of Life in Multiple Sclerosis: A Pre-test, Post-test Multicenter Implementation Study” by Robles-Sánchez *et al*.**

# Supplementary Tables

| **Table S1.** Study schedule to deliver questionnaires and assessments. | | | | | | | |
| --- | --- | --- | --- | --- | --- | --- | --- |
|  |  | **Intervention** | | | | **Follow-up** | |
|  | **Screening** | **S1** | **S2** | **S8** | **S9** | **6 m** | **12 m** |
| Sociodemographic data | ✔ |  |  |  |  |  |  |
| BICAMS (selection criterium) | ✔ |  |  |  |  |  |  |
| Knowledge questionnaire |  | ✔ |  | ✔ |  | ✔ | ✔ |
| HADS | ✔ |  |  | ✔ |  | ✔ | ✔ |
| Lifestyle and habits questionnaire |  | ✔ |  |  | ✔ | ✔ | ✔ |
| PAM-13 |  |  | ✔ |  | ✔ | ✔ | ✔ |
| Treatment compliance | ✔ |  |  |  |  |  | ✔ |
| Healthcare resources |  | ✔ |  |  |  |  | ✔ |
| MSQoL-54 |  | ✔ |  | ✔ |  | ✔ | ✔ |
| FSS |  |  | ✔ |  | ✔ | ✔ | ✔ |
| EDSS | ✔ |  |  |  |  |  | ✔ |
| Participant and EP experience |  |  |  |  | ✔ |  | ✔ |
| Attendance |  |  |  |  | ✔ |  |  |
| 6 m, 6 months; 12 m, 12 months; BICAMS, Brief International Cognitive Assessment for Multiple Sclerosis; EDSS, Expanded Disability Status Scale; EP, expert patient; FSS, Fatigue Severity Scale; HADS, Hospital Anxiety and Depression Scale; MSQoL-54, Multiple Sclerosis Quality of Life 54 items; PAM-13, Patient Activation Measure-13; SX, Session X. | | | | | | | |

| **Table S2.** Expert Patient Program Catalonia-Multiple Sclerosis (EPPC-MS): Sessions and contents | |
| --- | --- |
| **Session** | **Content description** |
| 1. Introduction | Presentation of group members, program objectives, session contents, session development, questionnaires |
| 2. The disease | What is multiple sclerosis? Concept, causes, types of MS, and diagnosis. |
| 3. Signs and symptoms | Flares (relapsing MS), progression (progressive MS), and fatigue |
| 4. Treatment | Existing treatment, future treatments, treatments during pregnancy, alternative therapies |
| 5. Health promotion | Healthy lifestyles: nutrition, activity and exercise, tobacco, vitamin D and sun exposure, obesity |
| 6. Taboo subjects | Urinary and fecal incontinency, sexuality and sexual dysfunction. |
| 7. Emotional impact and relationships | Coping with the disease, work and family relationships, available resources for support |
| 8. Social environment and quality of life |  |
| 9. Program finalization | Wrap-up session |

| **Table S3.** Questionnaire on disease-related knowledge. | |
| --- | --- |
| **Questions** | **Answers** |
| **Q1. In MS:** | a) The causes and mechanisms triggering the disease are multiple and complex. |
|  | b) An inflammation of the CNS occurs, mediated by our own immune system. |
|  | c) The main characteristic of the disease is demyelination (loss of the myelin that covers neurons). |
|  | d) All of the above are correct. |
| **Q2. The relapsing-remitting form of MS:** | a) Is the most frequent. |
|  | b) Is characterized by the presence of flares. |
|  | c) The symptoms of a flare usually develop over hours or days. |
|  | d) All of the above are correct. |
| **Q3. Currently, a MS diagnosis:** | a) Can only be established by MRI findings. |
|  | b) Can only be established by cerebrospinal fluid findings. |
|  | c) Can only be established through neurological exploration. |
|  | d) Is based on the McDonald diagnostic criteria. |
| **Q4. The McDonald criteria allow to establish a MS diagnosis:** | a) Even if MRI results are normal. |
|  | b) If the MRI shows presence of inflammatory plaques in at least two of the four characteristic locations for this disease and they are in different evolutionary stages (recent plaques and other old plaques). |
|  | c) If other alternative diagnoses that better explain the symptoms are ruled out. |
|  | d) Both b) and c) are correct. |
| **Q5. In progressive MS:** | a) Symptoms develop slowly and progressively over the years. |
|  | b) Flares are the more characteristic sign. |
|  | c) MRI is unnecessary for diagnosis because symptoms are unmistakable. |
|  | d) Testing cerebrospinal fluid is not useful in these cases. |
| **Q6. Diet and MS:** | a) No relationship exists between diet and MS. |
|  | b) A diet high in salt, fat, and red meat is recommended. |
|  | c) The Mediterranean diet is the recommended diet. |
|  | d) Gluten should be eliminated and carbohydrates reduced. |
| **Q7. Exercise and MS:** | a) Regular moderate exercise is recommended. |
|  | b) Physical exercise must be adapted to the abilities of the person with MS. |
|  | c) Exercise helps maintain a balanced body weight and reduces the risk of depression and anxiety. |
|  | d) All of the above are correct. |
| **Q8. Vitamin D, sun exposure, and MS:** | a) Vitamin D supplements are recommended only if the person has low levels and under medical prescription. |
|  | b) Two hours of sun exposure per day are recommended to produce and absorb vitamin D. |
|  | c) No relationship exists between sun exposure and vitamin D. |
|  | d) No relationship exists between sun exposure and MS. |
| **Q9. Smoking and MS:** | a) Smoking leads to worse prognosis and disease outcome. |
|  | b) Smoking is discouraged. |
|  | c) MS units can give advice on how to quit smoking. |
|  | d) All of the above are correct. |
| **Q10. Weight and MS:** | a) Being overweight worsens the progression of the disease. |
|  | b) Keeping an adequate weight is recommended, since it helps carry out the activities of daily living. |
|  | c) Regular exercise helps maintain an adequate weight. |
|  | d) All of the above are correct. |
| **Q11. MS and daily life abilities:** | a) It is crucial to know and value one’s abilities. |
|  | b) Abilities must be used wisely. |
|  | c) You must value and love yourself. |
|  | d) All of the above are correct. |
| **Q12. When MS appears:** | a) We do not accept the disease; we reject it. |
|  | b) It is a travel companion for life. |
|  | c) It affects everyone in a similar way. |
|  | d) The disease lasts a few months. |
| **Q13. Coping with stress and MS:** | a) A day-to-day plan is recommended. |
|  | b) Exercising is advisable to reduce stress. |
|  | c) You must learn to ask for help and be tolerant of yourself by learning to say NO. |
|  | d) All of the above are correct. |
| **Q14. Vitamin D and sun exposure in MS:** | a) Sunscreens prevent the synthesis of vitamin D. |
|  | b) Sunscreens help you receive vitamin D. |
|  | c) Vitamin D is not associated with the incidence of the disease. |
|  | d) None of the above are correct. |
| **Q15. Lifestyle and MS:** | a) Lifestyle has an impact on MS. |
|  | b) Smoking influences flare appearance. |
|  | c) Excessive salt intake can cause flares. |
|  | d) The introduction of physical exercise can cause flares. |
| **Q16. Urinary incontinence:** | a) When there is an urgency to urinate, an increase in frequency and/or a feeling of not emptying the bladder properly, you should consult with the healthcare team. |
|  | b) Urinary tract infections can increase fatigue and spasticity. |
|  | c) Bladder catheterization reduces the frequency of infections. |
|  | d) All of the above are correct. |
| **Q17. Fecal incontinence:** | a) Constipation is the most common intestinal disorder in people with MS. |
|  | b) The Mediterranean diet, drinking plenty of water and exercising can help avoid constipation. |
|  | c) Laxatives must be taken according to the instructions of professionals. |
|  | d) All of the above are correct. |
| **Q18. MS and sexual dysfunction:** | a) Sexual dysfunction may be related with the injuries and symptoms caused by the disease, and with psychological, educational, and sociocultural factors. |
|  | b) Some treatments, such as antidepressants, can cause sexual dysfunction. |
|  | c) Sexual dysfunction must be discussed openly within the couple and the treatment must be multidisciplinary. |
|  | d) All of the above are correct. |
| CNS, central nervous system; MRI, magnetic resonance imaging; MS, multiple sclerosis. | |

| **Table S4.** Distribution of participants into intervention groups in each participating unit | | |
| --- | --- | --- |
| **MS Unit** | **MS type** | **No. of participants** |
| Multiple Sclerosis Center of Catalonia (Cemcat), Vall d’Hebron Hospital (Barcelona) | Progressive | 10 |
|  | Relapsing | 8 |
| Joan XXIII University Hospital (Tarragona) & Sant Joan University Hospital (Reus) | Progressive | 8 |
|  | Relapsing | 10 |
| Neuroimmunology and Multiple Sclerosis Unit of Girona (Girona) | Progressive | 8 |
|  | Relapsing | 10 |
| MS-Neuroimmunology Unit of Hospital Clínic de Barcelona (Barcelona) | Progressive | 10 |
|  | Relapsing | 12 |
| Demyelinating Diseases Unit of the Germans Trias i Pujol University Hospital (Badalona) | Progressive | 10 |
|  | Relapsing | 7 |
| Arnau Vilanova University Hospital (Lleida) | Progressive | 9 |
|  | Relapsing | 10 |
|  | | |

| **Table S5.** Baseline sociodemographic and clinical characteristics of expert patients compared to participants. N=124 | | | |
| --- | --- | --- | --- |
|  | **Expert patients**  **n=12** | **Participants**  **n=112** | ***P*-value** |
| **Sociodemographic characteristics** |  |  |  |
| Sex, *n (%)* |  |  |  |
| Female | 10 (83.3) | 77 (68.8) | .5074^a^ |
| Male | 2 (16.7) | 35 (31.2) |  |
| Age (years), *median (IQR)* | 51.0 (48.0, 51.0) | 48.5 (42.0, 54.0) | .2623^b^ |
| Marital status, *n (%)* |  |  |  |
| Single, separated, divorced, widow(er) | 4 (33.3) | 24 (21.4) | .4655^a^ |
| Married, with partner | 8 (66.7) | 88 (78.6) |  |
| Support from relatives, *n (%)* |  |  |  |
| Yes | 12 (100) | 110 (98.2) | 1.0000^a^ |
| No | 0 (0) | 2 (1.8) |  |
| Educational level, *n (%)* |  |  |  |
| Primary school | 0 (0) | 21 (18.8) | .2402^a^ |
| High school and professional degree | 5 (41.7) | 44 (39.3) |  |
| Advanced studies (university, master’s, and doctorate) | 7 (58.3) | 47 (42) |  |
| Employment, *n (%)* |  |  |  |
| Employed | 7 (58.3) | 57 (50.9) | .6240^c^ |
| Retired, disability situation | 5 (41.7) | 55 (49.1) |  |
| **Clinical characteristics** |  |  |  |
| EDSS scores, *median (IQR)* | 3.75 (3.50, 6.00) | 4.00 (2.00, 6.00) | .4830^b^ |
| Family history of MS, *n (%)* |  |  |  |
| Yes | 0 (0) | 4 (3.6) | 1.0000^a^ |
| No | 12 (100) | 108 (96.4) |  |
| Disease-modifying treatment, *n (%)* |  |  |  |
| Yes | 12 (100) | 85 (75.9) | .0545^c^ |
| No | 0 (0) | 27 (24.1) |  |
| Type of disease-modifying treatment, *n (%)* |  |  |  |
| Self-administered | 6 (50) | 32 (37.6) | .5302^a^ |
| Hospital administered | 6 (50) | 53 (62.4) |  |
| EDSS, Expanded Disability Status Scale; IQR, interquartile range; MS, multiple sclerosis; SD, standard deviation.  ^a^Fisher's exact test  ^b^U Mann-Whitney test  ^c^Chi-squared test | | | |

| **Table S6.** Patients’ answers to the 18-item knowledge questionnaire at the indicated timepoints (only questions with non-statistically significant differences are shown), N (%). | | | | | |
| --- | --- | --- | --- | --- | --- |
|  | **S1** | **S8** | **6 m** | **12 m** | ***P*-value^a^** |
| **Q1. In MS:** | **N=112** | **N=108** | **N=101** | **N=106** | .095 |
| a)    The causes and mechanisms triggering the disease are multiple and complex. | 0 (0) | 1 (0.93) | 1 (0.99) | 4 (3.77) |  |
| b)   An inflammation of the CNS occurs, mediated by our own immune system. | 2 (1.79) | 1 (0.93) | 1 (0.99) | 1 (.94) |  |
| c)    The main characteristic of the disease is demyelination (loss of the myelin that covers neurons). | 19 (16.96) | 11 (10.19) | 10 (9.9) | 7 (6.6) |  |
| d)   All of the above are correct. | 91 (81.25) | 95 (87.96) | 89 (88.12) | 94 (88.68) |  |
| **Q4. The McDonald criteria allow establishing the MS diagnosis:** | **N=112** | **N=108** | **N=101** | **N=106** | .124 |
| a)      Even if MRI results are normal. | 0 (0) | 2 (1.85) | 0 (0) | 4 (3.77) |  |
| b)      If the MRI shows presence of inflammatory plaques in at least two of the four characteristic locations for this disease and they are in different evolutionary stages (recent plaques and other old plaques). | 39 (35.14) | 33 (30.56) | 23 (22.77) | 21 (19.81) |  |
| c)      If other alternative diagnoses that better explain the symptoms are ruled out. | 1 (.9) | 0 (0) | 2 (1.98) | 1 (.94) |  |
| d)      Both b) and c) are correct. | 71 (63.96) | 73 (67.59) | 76 (75.25) | 80 (75.47) |  |
| **Q5. In progressive MS:** | **N=112** | **N=108** | **N=101** | **N=106** | .055 |
| a)    Symptoms develop slowly and progressively over the years. | 92 (82.14) | 98 (90.74) | 82 (81.19) | 95 (89.62) |  |
| b)   Flares are the more characteristic sign. | 19 (16.96) | 9 (8.33) | 15 (14.85) | 5 (4.72) |  |
| c)    MRI is unnecessary for diagnosis because symptoms are unmistakable. | 1 (.89) | 0 (0) | 3 (2.97) | 3 (2.83) |  |
| d)   Testing cerebrospinal fluid is not useful in these cases. | 0 (0) | 1 (0.93) | 1 (0.99) | 3 (2.83) |  |
| **Q7. Exercise and MS:** | **N=112** | **N=108** | **N=101** | **N=106** | .419 |
| a)    Regular moderate exercise is recommended. | 2 (1.79) | 2 (1.85) | 2 (1.98) | 3 (2.83) |  |
| b)   Physical exercise must be adapted to the abilities of the person with MS. | 11 (9.82) | 5 (4.63) | 7 (6.93) | 7 (6.6) |  |
| c)    Exercise helps maintain a balanced body weight and reduces the risk of depression and anxiety. | 1 (.89) | 0 (0) | 0 (0) | 1 (.94) |  |
| d)   All of the above are correct. | 98 (87.5) | 101 (93.52) | 92 (91.09) | 95 (89.62) |  |
| **Q8. Vitamin D, sun exposure, and MS:** | **N=112** | **N=108** | **N=101** | **N=106** | .981 |
| a)    Vitamin D supplements are recommended only if the person has low levels and under medical prescription. | 99 (88.39) | 95 (87.96) | 89 (88.12) | 92 (86.79) |  |
| b)   Two hours of sun exposure per day are recommended to produce and absorb vitamin D. | 7 (6.25) | 10 (9.26) | 10 (9.9) | 11 (10.38) |  |
| c)    No relationship exists between sun exposure and vitamin D. | 2 (1.79) | 0 (0) | 0 (0) | 0 (0) |  |
| d)   No relationship exists between sun exposure and MS. | 4 (3.57) | 3 (2.78) | 2 (1.98) | 3 (2.83) |  |
| **Q9. Tobacco and MS:** | **N=112** | **N=108** | **N=101** | **N=106** | .123 |
| a)    Smoking leads to worse prognosis and disease outcome. | 4 (3.57) | 6 (5.56) | 4 (3.96) | 3 (2.83) |  |
| b)   Smoking is discouraged. | 14 (12.5) | 4 (3.7) | 4 (3.96) | 7 (6.6) |  |
| c)    MS units can give advice on how to quit smoking. | 0 (0) | 1 (.93) | 1 (.99) | 0 (0) |  |
| d)   All of the above are correct. | 94 (83.93) | 97 (89.81) | 92 (91.09) | 96 (90.57) |  |
| **Q13. Coping with stress and MS:** | **N=112** | **N=108** | **N=101** | **N=106** | .131 |
| a)    A day-to-day plan is recommended. | 1 (0.89) | 1 (0.93) | 2 (1.98) | 1 (.94) |  |
| b)   Exercising is advisable to reduce stress. | 0 (0) | 0 (0) | 0 (0) | 0 (0) |  |
| c)    You must learn to ask for help and be tolerant of yourself by learning to say NO. | 8 (7.14) | 4 (3.7) | 7 (6.93) | 2 (1.89) |  |
| d)   All of the above are correct. | 103 (91.96) | 103 (95.37) | 92 (91.09) | 103 (97.17) |  |
| **Q14. Vitamin D and sun exposure in MS:** | **N=112** | **N=108** | **N=101** | **N=106** | .587 |
| a)    Sunscreens prevent the synthesis of vitamin D. | 7 (6.25) | 16 (14.81) | 9 (8.91) | 11 (10.38) |  |
| b)   Sunscreens help you receive vitamin D. | 4 (3.57) | 2 (1.85) | 5 (4.95) | 2 (1.89) |  |
| c)    Vitamin D is not associated to the incidence of the disease. | 5 (4.46) | 2 (1.85) | 1 (.99) | 3 (2.83) |  |
| d)   None of the above are correct. | 96 (85.71) | 88 (81.48) | 86 (85.15) | 90 (84.91) |  |
| **Q15. Lifestyles and MS:** | **N=112** | **N=108** | **N=100** | **N=106** | .834 |
| a)   Lifestyle has an impact on MS. | 101 (90.18) | 95 (87.96) | 89 (89) | 94 (88.68) |  |
| b)   Smoking influences flare appearance. | 11 (9.82) | 11 (10.19) | 11 (11) | 10 (9.43) |  |
| c)    Excessive salt intake can cause flares. | 0 (0) | 2 (1.85) | 0 (0) | 2 (1.89) |  |
| d)   The introduction of physical exercise can cause flares. | 0 (0) | 0 (0) | 0 (0) | 0 (0) |  |
| 6 m, 6 months; 12 m, 12 months; CNS, central nervous system; MRI, magnetic resonance imaging; MS, multiple sclerosis; SX, Session X.  ^a^*P*-values were calculated using the Skillings-Mack test. | | | | | |

| **Table S7.** Patients’ answers to lifestyle habits test at the indicated timepoints. N (%) | | | | | | |
| --- | --- | --- | --- | --- | --- | --- |
|  | **S2** | **S9** | **6 m** | **12 m** | ***P*-value**^a^ |  |
| **Q1. Is your medication self- or hospital-administered?** | N= 111 | N=108 | N=100 | N=106 | **< .0001** |  |
| Hospital-administered | 53 (47.75) | 57 (52.78) | 56 (56.0) | 64 (60.38) |  |  |
| Self-administered (oral or self-injected) | 58 (52.25) | 51 (47.22) | 44 (44.0) | 42 (39.62) |  |  |
| **Q2. Do you take medication according to healthcare professionals' indications?*** | N=58 | N=51 | N=44 | N=42 | .223 |  |
| Always | 48 (82.76) | 49 (96.08) | 41 (93.18) | 37 (88.1) |  |  |
| Many times | 3 (5.17) | 0 (0) | 0 (0) | 2 (4.76) |  |  |
| Few times | 3 (5.17) | 1 (1.96) | 0 (0) | 1 (2.38) |  |  |
| Never | 4 (6.9) | 1 (1.96) | 3 (6.82) | 2 (4.76) |  |  |
| **Q3. Do you take medication at the same time?*** | N=58 | N=51 | N=44 | N=42 | .158 |  |
| Always | 36 (62.07) | 32 (62.75) | 24 (54.55) | 28 (66.67) |  |  |
| Many times | 21 (36.21) | 18 (35.29) | 17 (38.64) | 14 (33.33) |  |  |
| Few times | 0 (0) | 1 (1.96) | 1 (2.27) | 0 (0) |  |  |
| Never | 1 (1.72) | 0 (0) | 2 (4.55) | 0 (0) |  |  |
| **Q4. Do you ever forget to take the medication?*** | N=58 | N=51 | N=44 | N=42 | **.041** |  |
| Always | 0 (0) | 0 (0) | 0 (0) | 0 (0) |  |  |
| Many times | 0 (0) | 0 (0) | 0 (0) | 0 (0) |  |  |
| Few times | 33 (56.9) | 24 (47.06) | 18 (40.91) | 16 (38.1) |  |  |
| Never | 25 (43.1) | 27 (52.94) | 26 (59.09) | 26 (61.9) |  |  |
| **Q5. Do you take medication on your own initiative (self-medication)?** | N= 111 | N=108 | N=100 | N=106 | **.033** |  |
| Always | 4 (3.6) | 2 (1.85) | 2 (2) | 1 (.94) |  |  |
| Many times | 2 (1.8) | 4 (3.7) | 1 (1) | 4 (3.77) |  |  |
| Few times | 65 (58.56) | 63 (58.33) | 54 (54) | 49 (46.23) |  |  |
| Never | 40 (36.04) | 39 (36.11) | 43 (43) | 52 (49.06) |  |  |
| **Q6. When taking medication on your own initiative, who recommended it?** | n=111 | N=108 | N=100 | N=106 | .079 |  |
| Doctor/nurse | 42 (37.84) | 50 (46.3) | 34 (34) | 44 (41.51) |  |  |
| At the pharmacy | 17 (15.32) | 19 (17.59) | 14 (14) | 13 (12.26) |  |  |
| I do it based on my experience | 17 (15.32) | 9 (8.33) | 16 (16) | 8 (7.55) |  |  |
| I never take medication on my own initiative | 35 (31.53) | 30 (27.78) | 36 (36) | 41 (38.68) |  |  |
| **Q7. Which of the following statements best describes your tobacco behavior?** | n=111 | N=108 | N=100 | N=106 | .246 |  |
| Never smoked | 35 (31.53) | 35 (32.41) | 32 (32) | 37 (34.91) |  |  |
| Former smoker for >1 year | 54 (48.65) | 53 (49.07) | 52 (52) | 50 (47.17) |  |  |
| Former smoker <1 year | 3 (2.7) | 3 (2.78) | 2 (2) | 3 (2.83) |  |  |
| Currently considering quitting tobacco | 9 (8.11) | 10 (9.26) | 9 (9) | 7 (6.6) |  |  |
| Currently smoking >1 cigarette/cigar/pipe per day | 10 (9.01) | 7 (6.48) | 5 (5) | 9 (8.49) |  |  |
| **Q8. In the past week (Monday to Sunday), how many glasses or drinks did you take of…, *mean (SD)*** | n=111 | N=108 | N=100 | N=106 |  |  |
| 8.1 Beer (or similar)? | 0.91 (1.43) | 0.88 (1.42) | 0.77 (1.22) | 0.84 (1.34) | .833 |  |
| 8.2 Wine or champagne (or similar)? | 1.21 (2.21) | 1.12 (1.95) | 1.14 (2.45) | 0.98 (1.74) | .333 |  |
| 8.3 Cognac, carajillo, vermouth, liqueurs, or similar? | 0.15 (0.43) | 0.18 (0.77) | 0.11 (0.42) | 0.09 (0.35) | .314 |  |
| 8.4 Whiskey, gin, rum, aguardiente, cocktail or similar? | 0.15 (0.47) | 0.08 (0.31) | 0.04 (0.20) | 0.20 (0.72) | .500 |  |
| **Q9. Do you carry out any type of physical or sports activity in your free time?** | n=111 | N=108 | N=100 | N=106 | .918 |  |
| Daily | 26 (23.42) | 27 (25.00) | 24 (24.00) | 25 (23.58) |  |  |
| Three days a week | 25 (22.52) | 27 (25.00) | 23 (23.00) | 27 (25.47) |  |  |
| Less than three days a week | 10 (9.01) | 11 (10.19) | 9 (9.00) | 12 (11.32) |  |  |
| Less than one day a week | 12 (10.81) | 7 (6.48) | 8 (8.00) | 7 (6.60) |  |  |
| Occasionally | 20 (18.02) | 17 (15.74) | 20 (20.00) | 16 (15.09) |  |  |
| I do not exercise | 18 (16.22) | 19 (17.59) | 16 (16.00) | 19 (17.92) |  |  |
| **9.1 Type of activity** |  |  |  |  |  |  |
| Gymnastics | N=112 | N=112 | N=112 | N=112 | .264 |  |
| No | 83 (74.11) | 75 (66.96) | 83 (74.11) | 80 (71.43) |  |  |
| Yes | 29 (25.89) | 37 (33.04) | 29 (25.89) | 32 (28.57) |  |  |
| Dancing | N=112 | N=112 | N=112 | N=112 | .168 |  |
| No | 108 (96.43) | 109 (97.32) | 106 (94.64) | 110 (98.21) |  |  |
| Yes | 4 (3.57) | 3 (2.68) | 6 (5.36) | 2 (1.79) |  |  |
| Gardening | N=112 | N=112 | N=112 | N=112 | .668 |  |
| No | 104 (92.86) | 105 (93.75) | 106 (94.64) | 107 (95.54) |  |  |
| Yes | 8 (7.14) | 7 (6.25) | 6 (5.36) | 5 (4.46) |  |  |
| Walking the dog | N=112 | N=112 | N=112 | N=112 | .222 |  |
| No | 102 (91.07) | 100 (89.29) | 98 (87.5) | 104 (92.86) |  |  |
| Yes | 10 (8.93) | 12 (10.71) | 14 (12.5) | 8 (7.14) |  |  |
| Walking | N=112 | N=112 | N=112 | N=112 | .776 |  |
| No | 57 (50.89) | 59 (52.68) | 62 (55.36) | 61 (54.46) |  |  |
| Yes | 55 (49.11) | 53 (47.32) | 50 (44.64) | 51 (45.54) |  |  |
| Playing petanque | N=112 | N=112 | N=112 | N=112 |  |  |
| No | 112 (100) | 112 (100) | 112 (100) | 112 (100) |  |  |
| Yes | 0 (0) | 0 (0) | 0 (0) | 0 (0) |  |  |
| Other physical activities | N=112 | N=112 | N=112 | N=112 | **.028** |  |
| No | 78 (69.64) | 84 (75) | 93 (83.04) | 85 (75.89) |  |  |
| Yes | 34 (30.36) | 28 (25) | 19 (16.96) | 27 (24.11) |  |  |
| **9.2 For how long?** | N=93 | N=89 | N=84 | N=87 | .350 |  |
| 15 minutes | 9 (9.68) | 8 (8.99) | 12 (14.29) | 8 (9.2) |  |  |
| 15 - 30 minutes | 22 (23.66) | 22 (24.72) | 16 (19.05) | 21 (24.14) |  |  |
| 30 - 60 minutes | 47 (50.54) | 45 (50.56) | 45 (53.57) | 44 (50.57) |  |  |
| more than 60 minutes | 15 (16.13) | 14 (15.73) | 11 (13.1) | 14 (16.09) |  |  |
| **Q10. Do you get vaccinated against the flu?** | N=111 | n=108 | n=100 | n=106 | .921 |  |
| Yes | 45 (40.54) | 41 (37.96) | 39 (39) | 42 (39.62) |  |  |
| No | 66 (59.46) | 67 (62.04) | 61 (61) | 64 (60.38) |  |  |
| **Q11. Have you done any breathing and/or relaxation sessions?** | N=111 | n=108 | n=100 | n=106 | .478 |  |
| No | 50 (45.05) | 48 (44.44) | 51 (51) | 51 (48.11) |  |  |
| Yes | 61 (54.95) | 60 (55.56) | 49 (49) | 55 (51.89) |  |  |
| **Q12. Do you go to sleep at the same time every day?** | N=111 | n=108 | n=100 | n=106 | .286 |  |
| Always | 18 (16.22) | 19 (17.59) | 19 (19) | 26 (24.53) |  |  |
| Many times | 73 (65.77) | 68 (62.96) | 68 (68) | 66 (62.26) |  |  |
| Few times | 16 (14.41) | 20 (18.52) | 10 (10) | 12 (11.32) |  |  |
| Never | 4 (3.6) | 1 (.93) | 3 (3) | 2 (1.89) |  |  |
| **Q13. Approximately, do you sleep the same number of hours each day?** | N=111 | n=108 | n=100 | n=106 | .190 |  |
| Yes, usually | 92 (82.88) | 93 (86.11) | 78 (78) | 87 (82.08) |  |  |
| No, I am very irregular | 19 (17.12) | 15 (13.89) | 22 (22) | 19 (17.92) |  |  |
| **Q14. How many hours do you sleep daily?** *median* [IQR] | 7.5 (6.5, 8.0)  n=110 | 7.3 (6.5, 8.0)  n=108 | 7.5 (7.0, 8.0)  100 | 7.5 (7.0, 8.0)  n=106 | .152 |  |
| **Q15. Currently, do you continuously follow any special diet or regimen?** | N=111 | n=108 | n=100 | n=106 | .637 |  |
| Yes | 82 (73.87) | 85 (78.7) | 77 (77) | 79 (74.53) |  |  |
| No | 29 (26.13) | 23 (21.3) | 23 (23) | 27 (25.47) |  |  |
| **Q15.1 What kind of diet do you follow?** | N=29 | N=23 | N=23 | N=27 | **.004** |  |
| Fat-free | 2 (6.9) | 2 (8.7) | 3 (13.04) | 4 (14.81) |  |  |
| Salt free | 1 (3.45) | 1 (4.35) | 1 (4.35) | 1 (3.7) |  |  |
| Low sugar | 1 (3.45) | 2 (8.7) | 3 (13.04) | 3 (11.11) |  |  |
| Weight loss | 11 (37.93) | 7 (30.43) | 7 (30.43) | 10 (37.04) |  |  |
| Other | 14 (48.28) | 11 (47.83) | 9 (39.13) | 9 (33.33) |  |  |
| **Q16. Do you actively participate in family activities?** | N=111 | n=108 | n=100 | n=106 | .417 |  |
| Always | 42 (37.84) | 38 (35.19) | 42 (42) | 37 (34.91) |  |  |
| Many times | 49 (44.14) | 51 (47.22) | 37 (37) | 43 (40.57) |  |  |
| Few times | 18 (16.22) | 17 (15.74) | 17 (17) | 22 (20.75) |  |  |
| Never | 2 (1.8) | 2 (1.85) | 4 (4) | 4 (3.77) |  |  |
| **Q17. Do you participate in activities carried out in your community?** | N=111 | n=108 | n=100 | n=106 | .145 |  |
| Always | 4 (3.6) | 10 (9.26) | 12 (12) | 7 (6.6) |  |  |
| Almost always | 12 (10.81) | 6 (5.56) | 6 (6) | 12 (11.32) |  |  |
| Sometimes | 28 (25.23) | 35 (32.41) | 32 (32) | 31 (29.25) |  |  |
| Occasionally | 30 (27.03) | 25 (23.15) | 25 (25) | 31 (29.25) |  |  |
| Never | 37 (33.33) | 32 (29.63) | 25 (25) | 25 (23.58) |  |  |
| 6 m, 6 months; 12 m, 12 months; IQR, interquartile range (Q1-Q3). ^1^*P*-values were calculated using the Skillings-Mack test. Significant values (<0.05) are highlighted in bold. | | | | | | |

| **Table S8.** Number of primary care, emergency, and MS unit visits during the year before the intervention (session 1) and after the end of the intervention (12 months), *mean (SD)*. | | | |
| --- | --- | --- | --- |
|  | **Session 1 N=109 Relapsing, n=55 Progressive, n=54** | **12 months N=106 Relapsing, n=55 Progressive, n=51** | ***P*-value**^1^ |
| **Emergency care** |  |  |  |
| All participants | 0.45 (0.82) | 0.50 (1.06) | .7704 |
| Relapsing MS | 0.53 (0.90) | 0.55 (1.10) | .6262 |
| Progressive MS | 0.37 (0.73) | 0.45 (1.03) | .9553 |
| **MS unit visits** |  |  |  |
| All participants | 16.69 (28.89) | 13.30 (12.18) | .7325 |
| Relapsing MS | 22.22 (39.49) | 11.55 (8.35) | .1148 |
| Progressive MS | 11.06 (6.94) | 15.20 (15.14) | .2297 |
| **MS unit visits - type** |  |  |  |
| In-person |  |  |  |
| All participants | 11.32 (13.38) | 11.58 (11.77) | .9591 |
| Relapsing MS | 14.04 (17.70) | 9.80 (7.88) | .0911 |
| Progressive MS | 8.56 (5.49) | 13.49 (14.71) | .1217 |
| Remote |  |  |  |
| All participants | 5.26 (16.26) | 1.74 (2.37) | .1516 |
| Relapsing MS | 7.98 (22.48) | 1.76 (2.33) | .7704 |
| Progressive MS | 2.48 (2.91) | 1.71 (2.44) | .0583 |
| **MS unit visits - professional** |  |  |  |
| Neurologist |  |  |  |
| All participants | 4.10 (3.77) | 3.49 (2.58) | .8623 |
| Relapsing MS | 4.98 (4.50) | 3.85 (2.45) | .5914 |
| Progressive MS | 3.20 (2.60) | 3.10 (2.69) | .9152 |
| Nurse |  |  |  |
| All participants | 4.67 (3.82) | 3.73 (3.42) | **.0092** |
| Relapsing MS | 4.75 (4.34) | 3.85 (3.82) | .1401 |
| Progressive MS | 4.59 (3.24) | 3.59 (2.97) | **.0235** |
| Other professionals |  |  |  |
| All participants | 16.86 (41.84) | 5.81 (11.17) | **< .00001** |
| Relapsing MS | 13.04 (33.61) | 3.44 (7.33) | **< .00001** |
| Progressive MS | 20.76 (48.83) | 8.37 (13.82) | **.0031** |
| MS, multiple sclerosis; SD, standard deviation  ^1^*P*-values were calculated using a repeated measures ANOVA. Statistically significant *p*-values are shown in bold. | | | |

| **Table S9.** Satisfaction of study participants at session 9 and 12 months after the end of the intervention, N (%). | | | |
| --- | --- | --- | --- |
|  | **Session 9 N=108** | **12 months N=106** | ***P*-value** |
| **1. I felt comfortable with my group mates.** |  |  | .075 |
| 0 | 0 (0) | 0 (0) |  |
| 1 | 0 (0) | 0 (0) |  |
| 2 | 1 (.93) | 1 (.94) |  |
| 3 | 0 (0) | 1 (.94) |  |
| 4 | 7 (6.48) | 11 (10.38) |  |
| 5 | 100 (92.59) | 93 (87.74) |  |
| **2. Being part of this group has helped me understand how to care for myself.** |  |  | **.326** |
| 0 | 3 (2.78) | 1 (.94) |  |
| 1 | 0 (0) | 0 (0) |  |
| 2 | 4 (3.7) | 4 (3.77) |  |
| 3 | 10 (9.26) | 8 (7.55) |  |
| 4 | 22 (20.37) | 24 (22.64) |  |
| 5 | 69 (63.89) | 69 (65.09) |  |
| **3. The topics discussed were very important.** |  |  | .522 |
| 0 | 0 (0) | 0 (0) |  |
| 1 | 1 (.93) | 0 (0) |  |
| 2 | 2 (1.85) | 3 (2.83) |  |
| 3 | 1 (.93) | 2 (1.89) |  |
| 4 | 22 (20.37) | 20 (18.87) |  |
| 5 | 82 (75.93) | 81 (76.42) |  |
| **4. The topics covered were useful for my daily life.** |  |  | .575 |
| 0 | 1 (.93) | 0 (0) |  |
| 1 | 2 (1.85) | 0 (0) |  |
| 2 | 3 (2.78) | 3 (2.83) |  |
| 3 | 7 (6.48) | 8 (7.55) |  |
| 4 | 22 (20.37) | 24 (22.64) |  |
| 5 | 73 (67.59) | 71 (66.98) |  |
| **5. I found the schedule appropriate.** |  |  | .575 |
| 0 | 1 (.93) | 1 (.94) |  |
| 1 | 4 (3.7) | 1 (.94) |  |
| 2 | 4 (3.7) | 3 (2.83) |  |
| 3 | 11 (10.19) | 7 (6.6) |  |
| 4 | 25 (23.15) | 36 (33.96) |  |
| 5 | 63 (58.33) | 58 (54.72) |  |
| **6. I found the space used for the sessions suitable.** |  |  | .292 |
| 0 | 1 (.93) | 2 (1.89) |  |
| 1 | 2 (1.85) | 1 (.94) |  |
| 2 | 3 (2.78) | 3 (2.83) |  |
| 3 | 7 (6.48) | 8 (7.55) |  |
| 4 | 22 (20.37) | 23 (21.7) |  |
| 5 | 73 (67.59) | 69 (65.09) |  |
| **7. The expert patient explanations were clear.** |  |  | .130 |
| 0 | 0 (0) | 1 (.94) |  |
| 1 | 1 (.93) | 0 (0) |  |
| 2 | 1 (.93) | 1 (.94) |  |
| 3 | 3 (2.78) | 4 (3.77) |  |
| 4 | 10 (9.26) | 12 (11.32) |  |
| 5 | 93 (86.11) | 88 (83.02) |  |
| **8. I was comfortable talking about my situation in front of my group mates.** |  |  | .180 |
| 0 | 2 (1.85) | 0 (0) |  |
| 1 | 0 (0) | 0 (0) |  |
| 2 | 0 (0) | 2 (1.89) |  |
| 3 | 6 (5.56) | 7 (6.6) |  |
| 4 | 17 (15.74) | 26 (24.53) |  |
| 5 | 83 (76.85) | 71 (66.98) |  |
| **9. The attitude of the professional observers was appropriate.** |  |  | .244 |
| 0 | 0 (0) | 0 (0) |  |
| 1 | 1 (.93) | 0 (0) |  |
| 2 | 0 (0) | 0 (0) |  |
| 3 | 2 (1.85) | 1 (.94) |  |
| 4 | 7 (6.48) | 12 (11.32) |  |
| 5 | 98 (90.74) | 93 (87.74) |  |
| **10. The expert patient was able to awaken my interest in self-care.** |  |  | .127 |
| 0 | 1 (.93) | 1 (.94) |  |
| 1 | 4 (3.7) | 1 (.94) |  |
| 2 | 2 (1.85) | 2 (1.89) |  |
| 3 | 6 (5.56) | 5 (4.72) |  |
| 4 | 15 (13.89) | 17 (16.04) |  |
| 5 | 80 (74.07) | 80 (75.47) |  |
| **11. I identified with the expert patient.** |  |  | .107 |
| 0 | 0 (0) | 1 (.94) |  |
| 1 | 4 (3.7) | 2 (1.89) |  |
| 2 | 1 (.93) | 3 (2.83) |  |
| 3 | 7 (6.48) | 9 (8.49) |  |
| 4 | 18 (16.67) | 18 (16.98) |  |
| 5 | 78 (72.22) | 73 (68.87) |  |
| **12. I would repeat a similar experience about other topics.** |  |  | .382 |
| 0 | 0 (0) | 0 (0) |  |
| 1 | 2 (1.85) | 2 (1.89) |  |
| 2 | 2 (1.85) | 3 (2.83) |  |
| 3 | 6 (5.56) | 9 (8.49) |  |
| 4 | 20 (18.52) | 15 (14.15) |  |
| 5 | 78 (72.22) | 77 (72.64) |  |
| **13. I would recommend this experience to family or friends.** |  |  | **.015** |
| 0 | 0 (0) | 0 (0) |  |
| 1 | 1 (.93) | 1 (.94) |  |
| 2 | 1 (.93) | 2 (1.89) |  |
| 3 | 5 (4.63) | 5 (4.72) |  |
| 4 | 10 (9.26) | 15 (14.15) |  |
| 5 | 91 (84.26) | 83 (78.3) |  |
| ^1^*P*-values were calculated using the Skillings-Mack test. Statistically significant p-values are shown in bold.  0, not at all to 5, very much. | | | |

| **Table S10.** Satisfaction of expert patients at session 9 and 12 months after the end of the intervention, N (%). | | | |
| --- | --- | --- | --- |
|  | **Session 9 N=12** | **12 months N=12** | ***P*-value** |
| **1. My experience has been positive.** |  |  | N/A |
| 0 | 0 (0) | 0 (0) |  |
| 1 | 0 (0) | 0 (0) |  |
| 2 | 0 (0) | 0 (0) |  |
| 3 | 0 (0) | 0 (0) |  |
| 4 | 0 (0) | 0 (0) |  |
| 5 | 12 (100) | 12 (100) |  |
| **2. The knowledge provided by the professionals has been clear and explanatory.** |  |  | N/A |
| 0 | 0 (0) | 0 (0) |  |
| 1 | 0 (0) | 0 (0) |  |
| 2 | 0 (0) | 0 (0) |  |
| 3 | 0 (0) | 0 (0) |  |
| 4 | 0 (0) | 0 (0) |  |
| 5 | 12 (100) | 12 (100) |  |
| **3. The information and documentation provided were appropriate.** |  |  | 0.515 |
| 0 | 0 (0) | 0 (0) |  |
| 1 | 0 (0) | 0 (0) |  |
| 2 | 0 (0) | 0 (0) |  |
| 3 | 0 (0) | 0 (0) |  |
| 4 | 0 (0) | 2 (16.67) |  |
| 5 | 12 (100) | 10 (83.33) |  |
| **4. I found it complicated to lead a group for the first time.** |  |  | 0.298 |
| 0 | 3 (25) | 2 (16.67) |  |
| 1 | 1 (8.33) | 1 (8.33) |  |
| 2 | 3 (25) | 2 (16.67) |  |
| 3 | 5 (41.67) | 6 (50) |  |
| 4 | 0 (0) | 1 (8.33) |  |
| 5 | 0 (0) | 0 (0) |  |
| **5. I managed to create a good relationship between the group and me.** |  |  | 1 |
| 0 | 0 (0) | 0 (0) |  |
| 1 | 0 (0) | 0 (0) |  |
| 2 | 0 (0) | 0 (0) |  |
| 3 | 0 (0) | 0 (0) |  |
| 4 | 5 (41.67) | 6 (50) |  |
| 5 | 7 (58.33) | 6 (50) |  |
| **6. I would repeat the experience.** |  |  | N/A |
| 0 | 0 (0) | 0 (0) |  |
| 1 | 0 (0) | 0 (0) |  |
| 2 | 0 (0) | 0 (0) |  |
| 3 | 0 (0) | 0 (0) |  |
| 4 | 0 (0) | 0 (0) |  |
| 5 | 12 (100) | 12 (100) |  |
| N/A, not applicable  ^1^*P*-values were calculated using the Skillings-Mack test. Statistically significant *p*-values are shown in bold.  0, not at all to 5, very much. | | | |
